# Defects in the STIM1 SOARα2 domain affect multiple steps in the CRAC channel activation cascade

**DOI:** 10.1007/s00018-021-03933-4

**Published:** 2021-09-08

**Authors:** Carmen Höglinger, Herwig Grabmayr, Lena Maltan, Ferdinand Horvath, Heinrich Krobath, Martin Muik, Adela Tiffner, Thomas Renger, Christoph Romanin, Marc Fahrner, Isabella Derler

**Affiliations:** 1 Institute of Biophysics, Johannes Kepler University Linz, Gruberstrasse 40, 4020 Linz, Austria; 2 Institute of Theoretical Physics, Johannes Kepler University Linz, Altenbergerstraße 69, 4040 Linz, Austria

**Keywords:** STIM1, Orai1, OASF, CRAC channels, CAD, SOAR, Molecular dynamics, Protein-membrane interaction

## Abstract

The calcium release-activated calcium (CRAC) channel consists of STIM1, a Ca^2+^ sensor in the endoplasmic reticulum (ER), and Orai1, the Ca^2+^ ion channel in the plasma membrane. Ca^2+^ store depletion triggers conformational changes and oligomerization of STIM1 proteins and their direct interaction with Orai1. Structural alterations include the transition of STIM1 C-terminus from a folded to an extended conformation thereby exposing CAD (CRAC activation domain)/SOAR (STIM1-Orai1 activation region) for coupling to Orai1. In this study, we discovered that different point mutations of F394 in the small alpha helical segment (STIM1 α2) within the CAD/SOAR apex entail a rich plethora of effects on diverse STIM1 activation steps. An alanine substitution (STIM1 F394A) destabilized the STIM1 quiescent state, as evident from its constitutive activity. Single point mutation to hydrophilic, charged amino acids (STIM1 F394D, STIM1 F394K) impaired STIM1 homomerization and subsequent Orai1 activation. MD simulations suggest that their loss of homomerization may arise from altered formation of the CC1α1-SOAR/CAD interface and potential electrostatic interactions with lipid headgroups in the ER membrane. Consistent with these findings, we provide experimental evidence that the perturbing effects of F394D depend on the distance of the apex from the ER membrane. Taken together, our results suggest that the CAD/SOAR apex is in the immediate vicinity of the ER membrane in the STIM1 quiescent state and that different mutations therein can impact the STIM1/Orai1 activation cascade in various manners.

**Graphic abstract:**

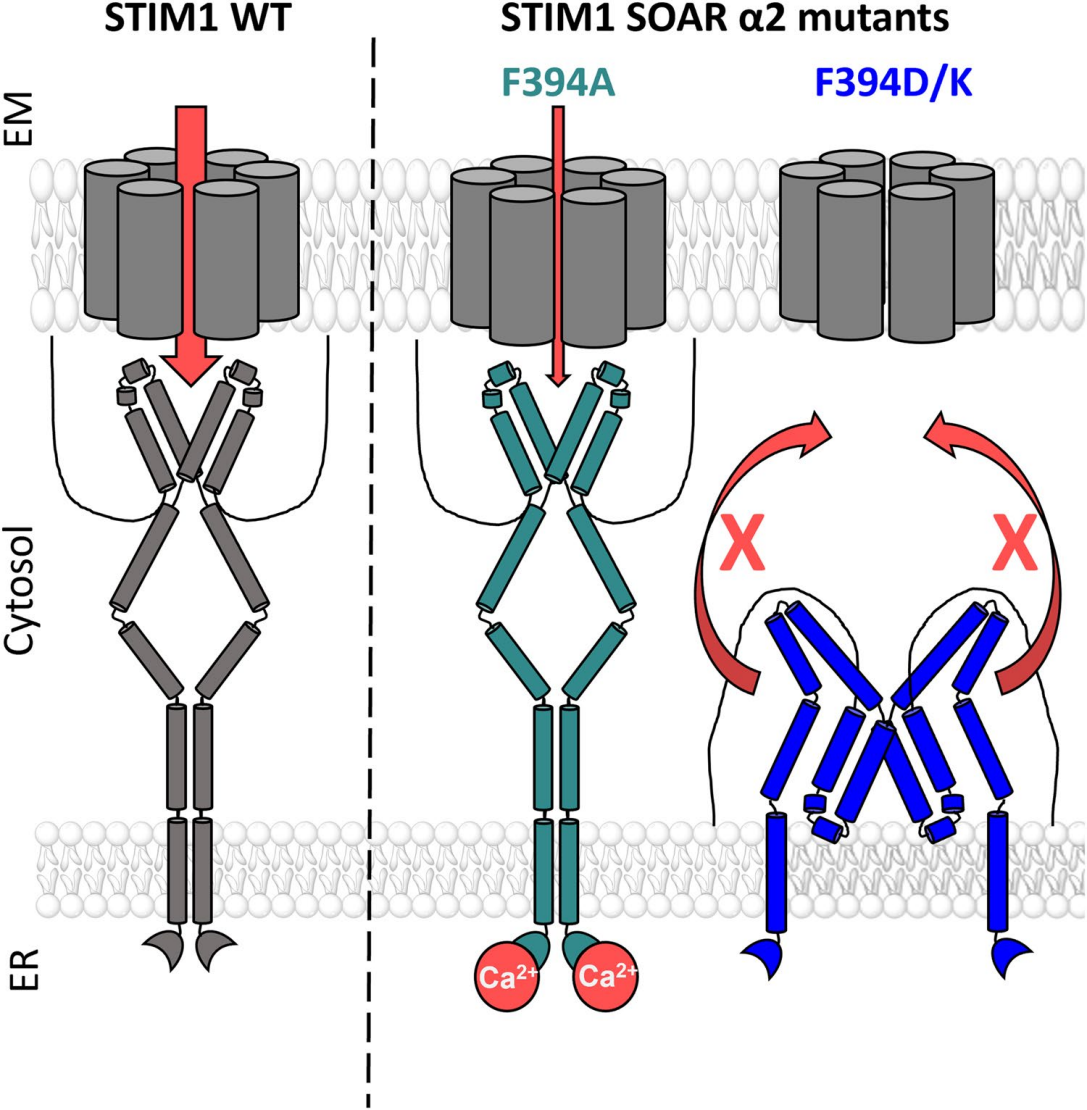

Legend: Upon intracellular Ca^2+^ store depletion of the endoplasmic reticulum (ER), Ca^2+^ dissociates from STIM1. As a result, STIM1 adopts an elongated conformation and elicits Ca^2+^ influx from the extracellular matrix (EM) into the cell due to binding to and activation of Ca^2+^-selective Orai1 channels (left). The effects of three point mutations within the SOARα2 domain highlight the manifold roles of this region in the STIM1/Orai1 activation cascade: STIM1 F394A is active irrespective of the intracellular ER Ca^2+^ store level, but activates Orai1 channels to a reduced extent (middle). On the other hand, STIM1 F394D/K cannot adopt an elongated conformation upon Ca^2+^ store-depletion due to altered formation of the CC1α1-SOAR/CAD interface and/or electrostatic interaction of the respective side-chain charge with corresponding opposite charges on lipid headgroups in the ER membrane (right).

**Supplementary Information:**

The online version contains supplementary material available at 10.1007/s00018-021-03933-4.

## Introduction

**Fig. 1:**
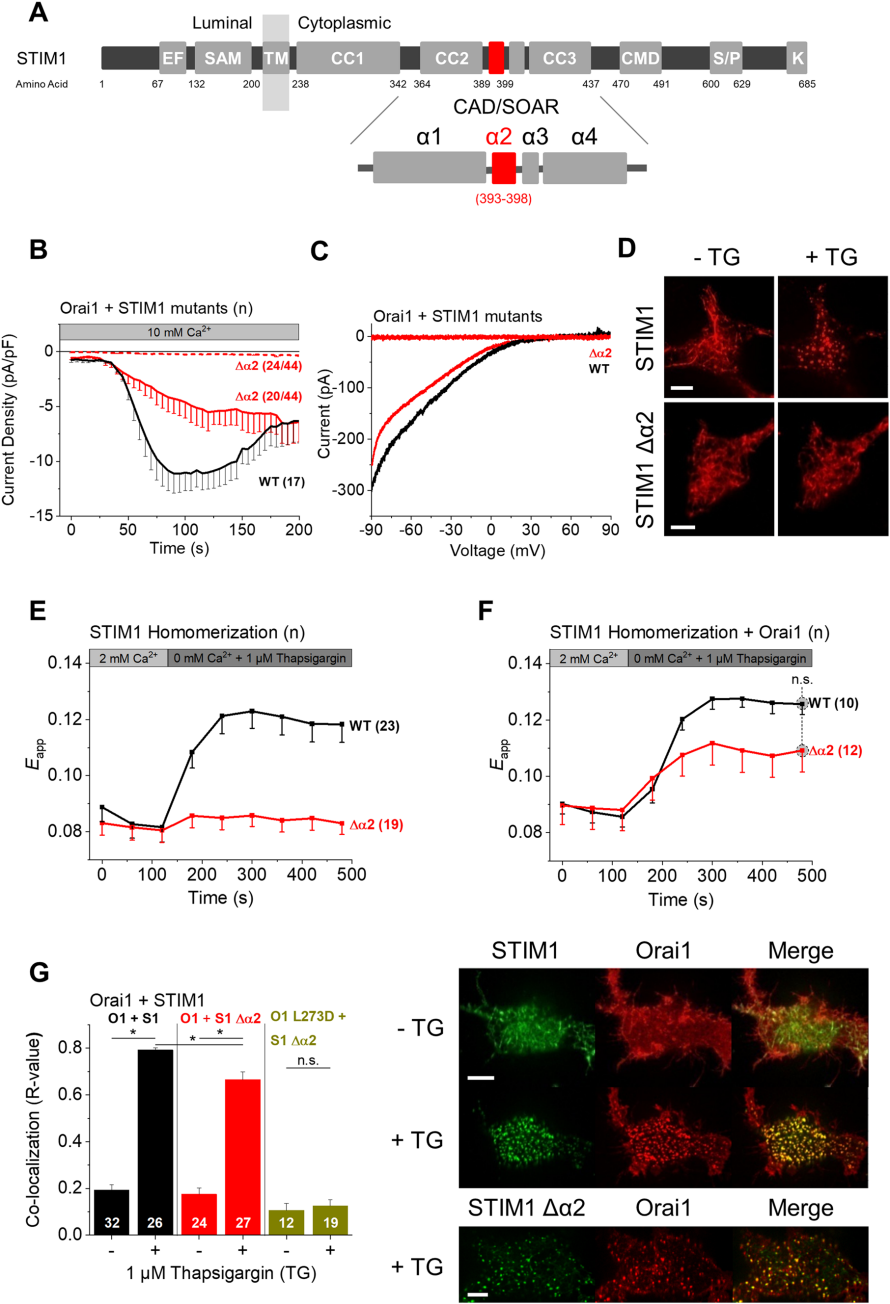
The STIM1 α2 domain affects STIM1 homomerization. **A** STIM1 domain structure with enlarged view of the CAD/SOAR fragment. The α2 domain (aa 393–398) is highlighted in red. **B** Time course of whole cell inward currents at − 74 mV activated by passive store depletion of HEK293 cells co-expressing Orai1 WT together with the following STIM1 constructs: STIM1 WT (wild-type) and Δα2 (Δaa 393–398). **C** Corresponding I/V relationships of maximum currents shown in (**B**). **D** Confocal fluorescence images of YFP-STIM1 WT and Δα2 before and after application of 1 µM TG. **E** Time course of FRET (*E*_app_) monitoring the homomerization of the respective CFP/YFP-labeled STIM1 constructs specified in (**B**) in response to application of 1 µM TG. **F** Same as in (**E**) but in the presence of Orai1 WT. **G** Left: calculated Pearson correlation coefficient (*R* value) as a quantitative measurement of co-localization between the indicated CFP-STIM1 mutants and YFP-Orai1 WT or L273D before and after treatment with 1 µM TG. The number *n* of cells is indicated within each bar. Right: confocal fluorescence images of representative cells (CFP in green, YFP in red) after treatment with 1 µM TG as well as an overlay (Merge in yellow) for visual comparison. Length of scale bars corresponds to 5 µm. Images were captured in the periphery of the cells. Data represent mean values ± SEM. HEK293 cells were used for all experiments. Student’s two-tailed *t* test was employed for statistical analyses with differences considered statistically significant at *p* < 0.05. Asterisks (*) indicate significant difference. Color code: STIM1 WT (black), STIM1 Δα2 (red), Orai1 L273D (yellow)

Store-operated calcium (SOC) entry is indispensable for a variety of cellular signaling processes such as gene expression, proliferation and apoptosis. The CRAC (Ca^2+^ release-activated Ca^2+^) channel is the most outstanding route for Ca^2+^ entry into the cell. Its activation is provoked by receptor-ligand binding at the plasma membrane (PM) which is followed by a signaling cascade resulting in the release of Ca^2+^ from the ER lumen. This release, in turn, triggers Ca^2+^ influx from the extracellular space into the cytosol [1]. The link between Ca^2+^ release from the ER and Ca^2+^ entry across the PM is accomplished by two molecular components: stromal interaction molecule 1 (STIM1), a Ca^2+^ sensor located in the ER membrane, and Orai1, a highly Ca^2+^ selective ion channel in the PM [2–7]. Under resting conditions in the cell, STIM1 proteins form dimers and are evenly spread within the ER membrane with robust linkage to microtubules [8, 9]. Upon Ca^2+^ store depletion, STIM1 is immediately activated, subsequently driven to ER-PM junctions where it interacts with and finally activates Orai1 by forming stable complexes, facilitating Ca^2+^ entry into the cell [10–15]. The choreography of STIM1 activation involves a series of essential steps such as STIM1 puncta formation through homomerization, and structural changes within the luminal, TM as well as a cytosolic domain, which altogether allow the formation of stable STIM1 oligomers [16–24]. An essential activation step within the C-terminus represents the release of the inhibitory clamp together with the exposure of the key communication site with Orai1, called SOAR (STIM1-Orai1 activation region, aa 344–442) [25] or CAD (CRAC activation domain, aa 342–448)[26], Fig. 1A). The CAD/SOAR region is sufficient for STIM1 oligomerization and Orai1 activation independent of store-depletion [16, 27]. It is composed of four helical regions termed α1, α2, α3 and α4 [28]. Direct interaction of CAD/SOAR with Orai1 C-terminus is an indispensable but not sufficient requisite for Orai1 pore opening [20, 29]. Additionally, α3 within this STIM1 C-terminal region directly interplays with a segment in the Orai1 loop2 close to TM3 to gate the channel [30], while the Orai1 N-terminus fine-tunes STIM1-mediated activation [26, 31–33].

In this study, we focus on the role of a small alpha helical segment, the α2 domain, within the CAD/SOAR region of STIM1 (Fig. 1A). Mutations in this region, in particular of F394, affect the tight, quiescent state of resting STIM1, either by causing partial constitutive activity (STIM1 F394A) or leading to loss-of-function (LoF) by strengthening the CC1 α1-SOAR/CAD binding interface and/or tethering the STIM1 apex to the ER membrane. Furthermore, our data suggest that α2 within the CAD/SOAR apex of resting STIM1 is oriented towards and close to the ER membrane.

## Methods

### Molecular cloning and mutagenesis

Human Orai1 (Orai1; accession no. NM_032790) was provided by A. Rao’s Laboratory, Harvard Medical School. N-terminally tagged Orai1 constructs were cloned with SalI and SmaI restriction sites of pECFP-C1 and pEYFP-C1 expression vectors (Clontech). Orai1 mutants (V102A, P245L, L273D) were generated using the QuikChange XL site-directed mutagenesis kit (Stratagene).

Human STIM1 (STIM1; accession no. NM_003156) N-terminally tagged with enhanced CFP (ECFP) and enhanced yellow fluorescent protein (EYFP) was provided by T. Meyer’s Laboratory, Stanford University. STIM1 C-terminus (amino acids 233–685) was cloned into the T/A site of pcDNA3.1V5 His TOPO by PCR and subcloned into pECFP-C1 and pEYFP-C1 via their internal restriction sites KpnI and XbaI. For the generation of cytosolic N-terminally tagged OASF a stop codon has been inserted at codon D475. All mutants (L251S, F394A, F394D, F394H, Δα2 (393–398), F394S, F394K, F394E, F394W, F394L, D475stop) were generated using the QuikChange XL site-directed mutagenesis kit (Stratagene). For double-tagged OASF constructs, CFP was cloned into pEYFP-C2 via SacII and Xba1 and the respective OASF (233–474) fragment was introduced via EcoRI and SacII. Constructs for the FIRE system consist of STIM1-signal peptide, EYFP (Y) or ECFP (C), 29 aa linker, STIM1 transmembrane domain, 32 glycine linker (GGGGSGGGGSEGKGGGGGGGGGFGGGGGGGGG) followed by protein fragment of interest [OASF (233–474), Orai1 N-terminus (1–90), Orai1 loop2 (137–184), Orai1 C-terminus (255–301)]. For experiments analyzing OASF α2 mutants’ ER membrane proximity, a 10 glycine linker (GGGGSGGGGS) as well as 0 glycine linker has been used, respectively. For generation of YFP- as well as CFP-STIM1 1–474 and 1–485, the respective stop codons at positions 475 and 486 have been inserted via point mutation. C-terminally tagged STIM1 was purchased from GeneCopoeiaTM (catalogue number EX-S0521-M02).

The integrity of all resulting clones was confirmed by sequence analysis.

### Cell culture and transfection

Human embryonic kidney 293 (HEK 293) cells were cultured in DMEM supplemented with l-glutamine (2 mM), streptomycin (100 μg/ml), penicillin (100 units/ml), and 10% fetal calf serum at 37 °C in a humidity-controlled incubator with 5% CO_2_. Transient transfection of HEK293 cells was performed using the TransFectin Lipid Reagent (Bio-Rad). Measurements were carried out 24 h following transfection. STIM1/STIM2 double knock-out HEK 293 cells were kindly provided by Matthias Hediger and Rajesh Bhardwaj and generated as described in Butorac et al. [30].

### Electrophysiology

Electrophysiological experiments were performed using the patch-clamp technique in whole-cell recording configurations at 21–25 °C. An Ag/AgCl electrode was used as reference electrode. Voltage ramps were applied every 5 s from a holding potential of 0 mV, covering a range of − 90 mV to + 90 mV over 1 s. Voltage step protocols were applied from a holding potential of 0 mV to − 70 mV for 1.5 s to determine FCDI. For passive store-depletion the internal pipette solution contained (in mM): 145 Cs methane sulfonate, 20 EGTA, 10 HEPES, 8 NaCl, 3.5 MgCl_2_, pH 7.2. Standard extracellular solution contained (in mM) 145 NaCl, 10 HEPES, 10 CaCl_2_, 10 glucose, 5 CsCl, 1 MgCl_2_, pH 7.4. Na^+^-DVF solution contained (in mM) 150 NaCl, 10 HEPES, 10 glucose, and 10 EDTA pH 7.4. A liquid junction potential correction of + 12 mV was applied, resulting from a Cl^−^-based bath solution and a sulfonate-based pipette solution. All currents were leak subtracted either by subtracting the initial voltage ramps obtained shortly following break-in with no visible current activation, or with constitutively active currents after application of 10 μM La^3+^ at the end of the experiment. All experiments were carried out at least on two different days.

### Confocal FRET fluorescence microscopy

Confocal FRET microscopy of HEK293 cells was carried out at 21–25 °C. The experimental setup consisted of a CSU-X1 Real-Time Confocal System (Yokogawa Electric Corporation, Japan) combined with two CoolSNAP HQ2 CCD cameras (Photometrics, AZ, USA). The installation was also fitted with a dual port adapter (dichroic: 505lp, cyan emission filter: 470/24, yellow emission filter: 535/30, Chroma Technology Corporation, VT, USA). An Axio Observer.Z1 inverted microscope (Carl Zeiss, Oberkochen, Germany) and two diode lasers (445 and 515 nm, Visitron Systems, Puchheim, Germany) were connected to the described configuration. All components were positioned on a Vision IsoStation anti-vibration table (Newport Corporation, CA, USA). Image recording and control of the confocal system were carried out with the VisiView software package (v2.1.4, Visitron Systems). Cross-excitation and spectral bleed-through necessitate image correction prior to any FRET calculation. Cross-excitation calibration factors were therefore determined for all expressed DNA constructs on each measurement day. After threshold determination as well as background signal subtraction, the apparent FRET efficiency *E*_app_ was calculated on a pixel-to-pixel basis. This was performed with a custom program [34] integrated into MATLAB (v7.11.0, The MathWorks, Inc., MA, USA) according to the method published by Zal, Gascoigne [35] with a microscope-specific constant *G* parameter of 2.75. The Pearson correlation coefficient (*R* value) was used to measure the strength of the linear association/co-localization between STIM1 and Orai1 variants, where a value of *R* = 1 signifies perfect positive correlation/co-localization. Experiments were replicated on at least two different days using independent transfections with the indicated number of cells (*n*).

### Molecular dynamics simulations

To obtain a model of the STIM1 quiescent state, we docked CC1α1 (PDB ID 6YEL, [19]) to the CAD/SOAR domain (PDB ID 3TEQ, [28]) using HADDOCK [36] (after restoring the WT sequence of the CAD/SOAR structure). Based on interaction sites identified in reference [37], residues 258, 261, 416, 419 and 423 were designated as active residues for HADDOCK ambiguous interaction restraints. From the resultant clusters, structures exhibiting incorrect CC1α1-CAD/SOAR orientation or involving steric clashes with the membrane were discarded. We proceeded using the best-scoring structure from among the remaining clusters (see Supp. Figure 1 and Table 1 for details). In the CC1 NMR model, the CAD/SOAR binding site is occupied by helices CC1α2,3. To allow for CAD/SOAR binding, we unfolded the NMR model with a bias potential by raising the CA atom RMSD and the radius of gyration while keeping helical secondary structure elements intact. Then the CAD/SOAR domain was appended to the unfolded CC1 model and the CC1α1-CAD/SOAR interface predicted by HADDOCK was established using targeted MD [38]. The docked structure was equilibrated for about 200 ns. We then added the TM domain, which was modeled as a helix based on the AA sequence with the help of MODELLER [39]. Electrostatic potentials were computed with TAPBS [40] and protonation states were assigned according to protonation patterns sampled using KARLSBERG 2.0 [41]. Using CHARMM GUI [42, 43], the combined system (residues 214–443) was embedded in a membrane consisting of DDPC and DLPE (with a ratio of 2:1), which are the two most abundant polar lipids in the mammalian ER membrane [44]. The overall lipid patch size was 90 × 90 Å^2^. The system was neutralized using 0.15 M KCl. For subsequent simulations of F394X (X = A, H, F, D, K), simulation boxes were neutralized by adding 4–8 Cl ions. F394D embedded in a phosphatidylserine (PS) membrane was prepared by using the same settings and changing only the membrane composition. The systems were heated and equilibrated using the standard CHARMM GUI six-step process combined with an additional unrestrained equilibration run. The mutants were modeled with CHARMM GUI using the same procedure as described above.

**Table 1:**
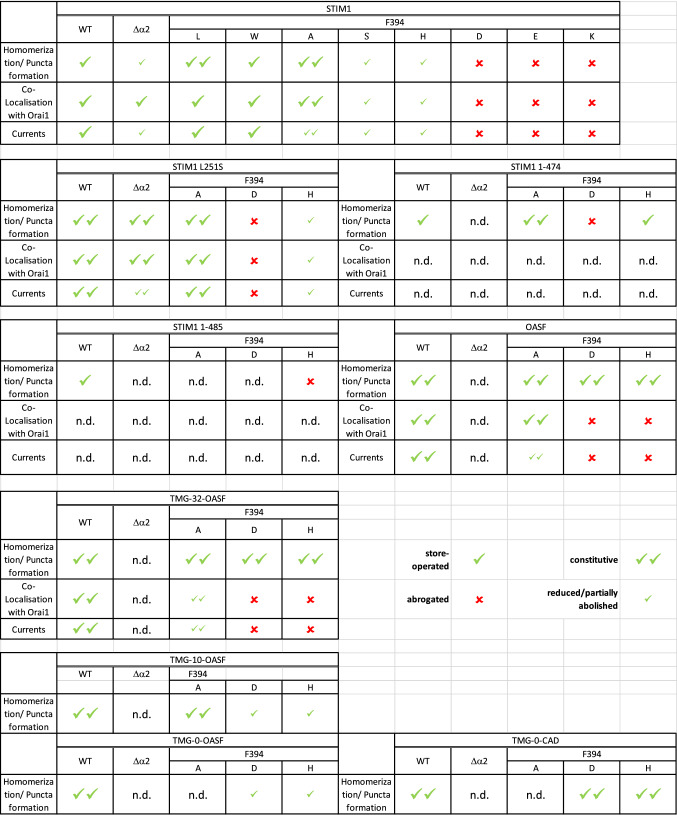
Overview of STIM1 α2 mutations and their effects on diverse steps in the STIM1/Orai1 activation cascade

Effects of α2 mutations in STIM1, STIM1 L251S and OASF on their ability to homomerize/form puncta, colocalize with Orai1 as well as induce CRAC channel currents. Green check marks indicate whether the different steps are activated in a store-operated manner (1 check mark), constitutive manner (2 check marks) and whether they are reduced or partially abolished (small check marks). Red crosses indicate loss of function of the respective step (n.d. not determined)

The WT, F394D and F394K were simulated for 1 μs at 0.15 M KCl. For each run, the final 500 ns were used for data analysis. For the neutralized simulations of F394X (X = A, H, F, D, K), total run times were between 280 and 360 ns. Here, after sufficient equilibration, the final 200 ns were used for data analysis. All MD simulations were produced using NAMD 2.13 [45] with the CHARMM36m force field [46]. Equilibration and structural stability of the constructs is demonstrated in Supp. Figure 2. The NpT ensemble was employed with a constant temperature of 300 K using Langevin dynamics. Pressure was set to 1 atm with the Langevin piston method. Long range electrostatic interactions were handled with the particle-mesh Ewald method. Dynamics were calculated using the velocity Verlet algorithm with an integration time step of 2 fs. H-bonds were restrained using ShakeH. Trajectories were analyzed with pytraj [47, 48] and mdtraj [49]. Interaction energies were calculated with CHARMM [50] and gRINN [51] using standard settings for pair list cutoffs and switching functions, in line with those used by NAMD. Lipid headgroup density was calculated by counting the positions of lipid N atoms in a grid with a spacing of 0.25 Å after adding 8 adjacent PBC images and aligning trajectory frames.

### Statistics

Live-cell results are depicted as mean values ± SEM determined for the indicated number *n* of experiments. For statistical comparisons, the Student’s two-tailed *t *test together with the *F* test of equality of variances was applied, considering differences statistically significant at *p* < 0.05. The one-sample Kolmogorov–Smirnov test was used to verify the presence of a normal distribution for the analyzed datasets. The Grubbs test was applied to eliminate outliers.

## Results

### Deletion of the STIM1 α2 domain affects STIM1 homomerization

The cytosolic portion of STIM1 and its molecular choreography evoking activation of Orai1 channels is a subject of high interest. CAD/SOAR represents the irreducible domain required for Orai1 activation [25, 26] and is mainly composed of four alpha helical domains (α1, α2, α3, α4) [28] (Fig. 1A). The roles of α1 and α4 have been investigated in detail for quite some time [20, 27, 52–54]. We recently reported that α3 is critical for an interplay with the cytosolic Orai1 loop2 to relay the gating signal to the pore [30]. However, the function of the well-conserved α2 domain (amino acids (aa) 393–398) is much less understood. It has been reported [55] that a single point mutation within the α2 domain (F394H) leads to loss of Orai1 activation. To further characterize the role of F394 together with the overall impact of the α2 domain, we initially deleted the entire α2 region in STIM1 (STIM1 Δα2, STIM1 Δ393-398) including F394 to examine the effect on STIM1 function and Orai1 channel activation. Patch clamp recordings revealed that STIM1 Δα2 was able to induce Orai1 currents with a similar reversal potential (V_rev_) as obtained with STIM1-WT. However, STIM1 Δα2 mediated Orai1 activation was observed in only ~ 50% of the experiments (Fig. 1B, C). Orai1 activation occurred more slowly and did not reach maximum current levels as seen for STIM1 WT (Fig. 1B). Analogously, we also detected in STIM1/STIM2 double knock-out (S1/S2 dKO) HEK 293 cells only in 50% of the experiments STIM1 Δα2-mediated Orai1 currents. However, Orai1 current activation was reduced by 80% compared to WT levels, suggesting that endogenous STIM proteins support the activity of STIM1 Δα2 via potential formation of heteromers (Supp. Figure 3A). Additionally, STIM1 Δα2, when expressed alone, failed to form characteristic puncta following store depletion (Fig. 1D). This was most likely caused by a substantial impairment of homo-/oligomerization, as evident from confocal FRET microscopy (Fig. 1E). Interestingly, homomerization of STIM1 Δα2 was partially rescued after store depletion when co-expressed with Orai1 (Fig. 1F). We observed a reduced but still pronounced co-localization of STIM1 Δα2 with Orai1 (Fig. 1G). This co-localization is abolished upon the introduction of the L273D mutation in Orai1, which is known to disrupt the initial binding of STIM1 to Orai1 [56, 57] (Fig. 1G).

To assess whether a partially open Orai1 conformation facilitates or even fully restores store-dependent activation via STIM1 Δα2, we investigated the effect of STIM1 Δα2 on the constitutive Orai1 P245L mutant [58], which is associated with the Stormorken-like syndrome [59]. Indeed, constitutive Orai1 P245L currents were further enhanced upon passive store-depletion in the presence of STIM1 Δα2 (Supp. Figure 3C), likely due to store-dependent coupling. Moreover, co-localization of STIM1 Δα2 with Orai1 P245L upon store-depletion reached similar levels as with Orai1 WT (Supp. Figure 3D). To examine the extent to which STIM1Δα2 mediated Orai1 activation differs from that induced by STIM1 WT, we further analyzed the authentic CRAC channel hallmarks [60, 61]. These included three types of typical biophysical characteristics of CRAC channels: (1) the V_rev_, which is approximately +50 mV, (2) the rapid Ca^2+^-dependent inactivation within 50 ms of an applied voltage step from 0 mV to very negative voltages (e.g. − 70 mV) and, (3) the increase in divalent-free Na^+^ containing versus Ca^2+^ containing currents. We discovered that these authentic CRAC channel hallmarks were affected (Table 2). STIM1 Δα2-Orai1 P245L currents exhibited no fast calcium dependent inactivation (FCDI), but only reactivation as typically observed for a variety of constitutive Orai1 mutants in the absence of STIM1 [60] (Supp. Figure 3E). Moreover, typical enhancements of currents in a divalent-free Na^+^ containing (I_DVF_) compared to Ca^2+^ containing solution (I_Ca2+_) as known for WT STIM1/Orai1 currents [60] were less pronounced (Supp. Figure 3F). Thus, STIM1 Δα2 is impaired in the communication with both Orai1 as well as Orai1 P245L. Moreover, we used the constitutive Orai1 V102A mutant as it can be employed to detect correct STIM1 binding based on the reversal potential of its current. It is non-selective in the absence of STIM1, but regains Ca^2+^ selectivity in the presence of STIM1, as visible via a right-ward shift of the V_rev_ to ~  +50 mV [32, 62]. STIM1 Δα2 was unable to enhance V_rev_ of the Orai1 V102A mutant which is in support of its defective communication with Orai1 channels [32, 62] (Supp. Figure 3G; Table 2). This indicates that STIM1 Δα2 somewhat faultily couples to Orai1.

**Table 2:** Overview of the typical CRAC channel hallmarks of STIM1 mutants

	Without STIM1	STIM1	STIM1 Δα2	STIM1 F394A	STIM1 F394H	STIM1 F394D
V_rev_ [mV]						
Orai1	–	59.22 ± 5.92	43.85 ± 4.35	57.00 ± 5.59	55.60 ± 7.45	–
Orai1 P245L	33.4 ± 1.88	40.16 ± 5.3	46.52 ± 9.81	41.39 ± 5.69	40.05 ± 9.39	43.27 ± 1.98
Orai1 V102A	32.58 ± 1.85	49.31 ± 3.31	31.63 ± 4.71	46.70 ± 10.98	58.10 ± 2.97	27.41 ± 4.83
FCDI						
Orai1 P245L	–	Yes	No	Yes	No	No
I_DVF_:I_Ca_^2+^						
Orai1 P245L	0.5	5.5	2.7	2.1	0.6	2.2

Table summarizes the V_rev_, FCDI and the ratio I_DVF_:I_Ca_^2+^ of Orai1 and Orai1 P245L currents in the absence of STIM1 and the presence of STIM1, STIM1 Δα2, STIM1 F394A, STIM1 F394H and STIM1 F394D. Moreover, the V_rev_ of Orai1 V102A currents in the absence of STIM1 and the presence of STIM1, STIM1 Δα2, STIM1 F394A, STIM1 F394H and STIM1 F394D are listed

In summary, impaired STIM1 Δα2 homomerization is presumably compensated by the coupling of STIM1 Δα2 to Orai1 to a certain degree. This facilitates STIM1 Δα2 puncta formation as well as co-localization with Orai1. Although Orai1 P245L promoted coupling of and activation by STIM1 Δα2, authentic CRAC channel hallmarks were not maintained. In support of constrained activity of STIM1 Δα2, also constitutive Orai1 V102A currents remained non-selective in the presence of this STIM1 mutant.

### STIM1 F394 is a critical residue within the α2 domain

**Fig. 2:**
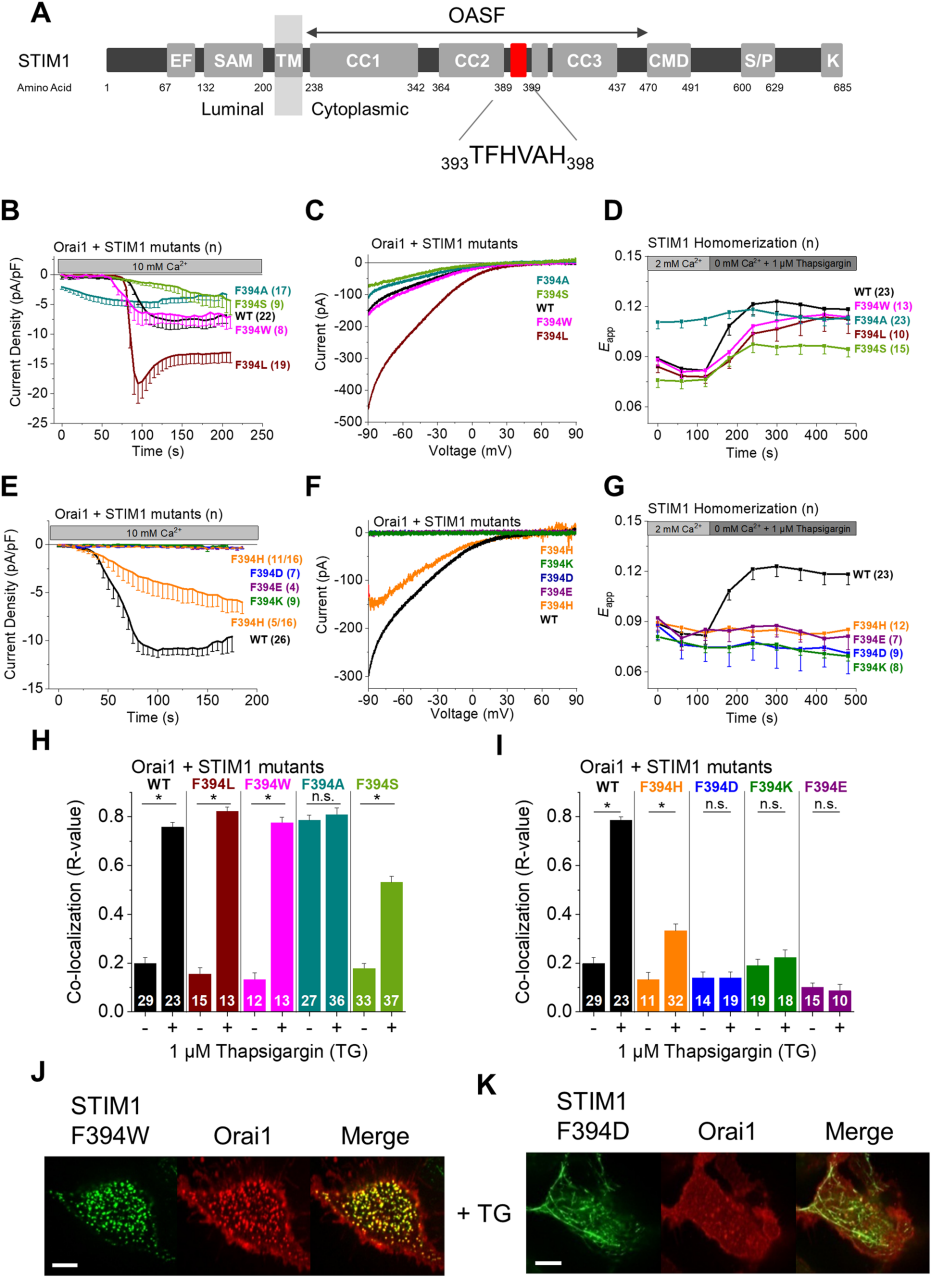
STIM1 F394 is a critical residue within the α2 domain. **A** STIM1 domain structure with the α2 domain (aa 393–398) highlighted in red. The OASF domain as well as the α2 amino acid sequence are indicated. **B**, **E** Time course of whole cell inward currents at − 74 mV activated by passive store depletion of HEK293 cells co-expressing Orai1 WT together with the following STIM1 constructs: STIM1 WT, F394W, F394L, F394A, F394S, F394D, F394E, F394H, and F394K. **C**, **F** Corresponding I/V relationships of maximum currents shown in (**B**) and (**E**). **D**, **G** Time course of FRET (*E*_app_) monitoring the homomerization of the respective CFP/YFP-labeled STIM1 constructs specified in (**B**) and (**E**) in response to 1 µM TG. **H**, **I** Calculated Pearson correlation coefficient (*R* value) as a quantitative measurement of co-localization between the indicated CFP-STIM1 mutants and YFP-Orai1 WT before and after treatment with 1 µM TG. The number n of cells is indicated within each bar. **J**, **K** Confocal fluorescence images of representative mutants (CFP in green, YFP in red) after treatment with 1 µM TG as well as an overlay (Merge in yellow) for visual comparison. Length of scale bars corresponds to 5 µm. Images were captured in the periphery of the cells. Data represent mean values ± SEM. HEK293 cells were used for all experiments. Student’s two-tailed *t* test was employed for statistical analyses with differences considered statistically significant at *p* < 0.05. Asterisks (*) indicate significant difference. Color code: STIM1 WT (black), F394A (dark cyan), F394D (blue), F394E (purple), F394H (orange), F394K (dark green), F394L (brown), F394W (magenta), F394S (light green)

Previous studies narrowed down that the only non-conserved residue F394 within the α2 domain of STIM1 is critical for the functional differences of STIM1 and STIM2 SOAR (Fig. 2A) [55]. Thus, we investigated its specific role and introduced various single point mutations at this position, including a selection of hydrophobic amino acids of distinct size (W, L, A), a hydrophilic residue (S) and charged residues (D, K, H).

Substitution to other hydrophobic amino acids (STIM1 F394L, STIM1 F394W) left store-dependent Orai1 current activation and STIM1 mutant homomerization as well as co-localization with Orai1 largely unaffected (Fig. 2B–D, H, J; Supp. Figure 4A). It is of note that store-operated Orai1 activation by STIM1 F394L led to almost doubled maximum currents compared to WT (Fig. 2B, C) which interestingly was not observed in S1/S2 dKO HEK 293 cells (Supp. Figure 3A). The mutation of F394 to a hydrophobic, but much smaller amino acid (STIM1 F394A) has been previously reported to decrease Orai1 activation compared to STIM1 WT [55]. Unexpectedly, STIM1 F394A led to gain-of-function (GoF) as visible via constitutive STIM1 homomerization as well as STIM1-Orai1 coupling independent of store-depletion to similar levels as obtained after store-depletion (Fig. 2D, H; Supp. Figure 4A). However, constitutive, maximum Orai1 currents, reached in the presence of STIM1 F394A, were reduced compared to STIM1 WT (Fig. 2 B, C).

The substitution to a serine (STIM1 F394S) led to store-operated Orai1 activation, homomerization and co-localization with Orai1. However, all these steps in the STIM1 F394S/Orai1 signaling cascade were consistently reduced compared to STIM1 WT (Fig. 2B–D, H).

In contrast, replacement to charged or more hydrophilic amino acids (STIM1 F394D, STIM1 F394E, STIM1 F394K, STIM1 F394H) abolished or drastically reduced Orai1 current activation (Fig. 2E–G, I). These STIM1 mutants unexpectedly lacked homomerization (Fig. 2G; Supp. Figure 4B). Thus, it cannot be concluded that there is a simple coupling defect with Orai1 since these mutations already affect an earlier step of the STIM1 activation cascade. Notably, the results for STIM1 F394H were less clear in our whole-cell recordings, as this mutant was able to induce store-dependent Orai1 current activation in ~ 30% of the experiments, although to a lower extent compared to STIM1 WT (Fig. 2E, F). We observed analogous functional effects in S1/S2 dKO HEK 293 cells (Supp. Figure 3A). STIM1 F394H revealed no homomerization in the absence of Orai1 (Fig. 2G). In the presence of Orai1, STIM1 F394H showed a slight increase in homomerization FRET in about 50% of the cells (Supp. Figure 4B). In line, co-localization of STIM1 F394H with Orai1 was significantly enhanced upon store-depletion, but was drastically reduced compared to STIM1 WT (Fig. 2I), although their FRET (YFP-Orai1 + STIM1 F394H-CFP) was not enhanced upon store-depletion (Supp. Figure 4C). The latter may be due to a combined effect of reduced affinity of the histidine mutant to Orai1 together with a potential perturbing effect of the C-terminal fluorescence labels. STIM1 F394D, STIM1 F394K and STIM1 F394E showed no increase in homomerization upon store depletion, concomitant with a lack of co-localization with Orai1 (Fig. 2G, I, K; Supp. Figure 4B). As a representative of inactive F394 mutants, STIM1 F394D–CFP showed no enhancement in FRET with YFP-Orai1, in accordance with the co-localization studies (Supp. Figure 4C).

This striking behavior was further challenged by the use of 2-APB that is known to act as a transient booster of STIM1-Orai1 coupling and CRAC currents [63]. Patch clamp experiments revealed that 75 µM 2-APB was able to evoke a substantial Orai1 current increase for both STIM1 WT as well as STIM1 F394H but not for STIM1 F394D (Supp. Figure 5A). Correspondingly, 2-APB increased homomerization as well as co-localization with Orai1 for STIM1 F394H, reaching levels comparable to STIM1 WT, but not for STIM1 F394D (Supp. Figure 5B–D).

Electrophysiological investigation of the ability of the STIM1 F394X (X = A, D, H) mutants to further activate Orai1 P245L revealed that STIM1 F394H enhanced small, constitutive activity of Orai1 P245L in a store-dependent manner, although not to the same maximum level as obtained with STIM1 WT (Supp. Figure 3C). STIM1 F394A and STIM1 F394D did not significantly enhance constitutive activity of Orai1 P245L (Supp. Figure 3C). Upon store-depletion, co-localization of STIM1 F394A or STIM1 F394H with Orai1 P245L reached levels comparable to those observed with STIM1 WT (Supp. Figure 3D). STIM1 F394D was unable to co-localize with Orai1 P245L similar like with Orai1 WT (Supp. Figure 3D). Of all STIM1 F394X (X = A, D, H) mutants, only STIM1 F394A co-expressed with Orai1 P245L retained an FCDI comparable to that of STIM1 WT + Orai1 P245L currents (Supp. Figure 3E). Enhancements in I_DVF_ versus I_Ca2+_ were reduced for all STIM1 F394X (X = A, D, H) mutants compared to STIM1 WT (Supp. Figure 3F). While STIM1 F394H and STIM1 F394A were able to restore Ca^2+^ selectivity of Orai1 V102A, STIM1 F394D failed to do so (Supp. Figure 3G; Table 2).

In summary, different amino acid substitutions of F394 in the α2 region led either to GoF or LoF. We discovered that these mutations entail multifaceted effects on STIM1 homomerization, co-localization with and activation of Orai1. Furthermore, they affect the maintenance of typical CRAC channel features either partially or completely, which is indicative of faulty or missing STIM1 coupling, respectively. Thus, the α2 domain does not only affect the gating of the Orai1 channel, but plays a more complex regulatory role in the STIM1/Orai1 activation machinery.

### F394 does not impact the conformational switch of the cytosolic STIM1 C-terminal fragment

**Fig. 3:**
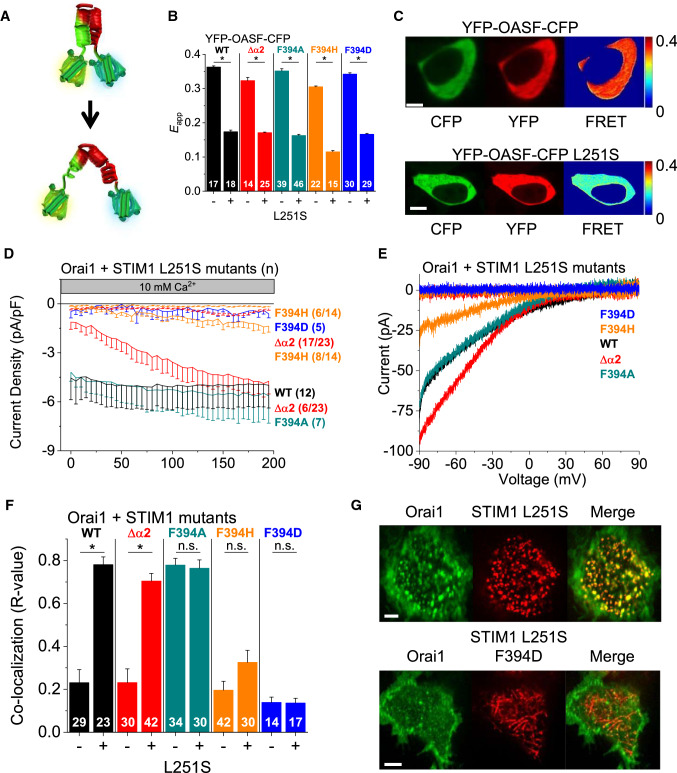
F394 does not impact the conformational switch of the STIM1 OASF fragment. **A** Simplified depictions of YFP-OASF-CFP conformational sensor in a tight, quiescent (top) and an extended, open (bottom) state. **B** Intramolecular FRET (*E*_app_) measurements of YFP-OASF-CFP conformational sensor probing the effect of the L251S mutation on the following constructs: OASF WT, Δα2 (Δaa 393–398), F394A, F394D, and F394H. The number n of cells is indicated within each bar. **C** Confocal fluorescence images of representative cells (CFP in green, YFP in red) as well as a FRET image showing calculated (*E*_app_) on a pixel-to-pixel basis (orange = high FRET, blue = low FRET). Images were captured in the center of the cells, rendering the outline of the nuclei visible. **D** Time course of whole cell inward currents at − 74 mV of HEK293 cells co-expressing Orai1 WT together with the following STIM1 constructs: STIM1 L251S, L251S Δα2, L251S F394A, L251 F394D, and L251S F394H. **E** Corresponding I/V relationships of maximum currents shown in (**D**). **F** Calculated Pearson correlation coefficient (*R* value) as a quantitative measurement of co-localization between CFP-Orai1 WT and the indicated YFP-STIM1 mutants (corresponding to those in (**B**) in the presence and absence of the L251S mutation. The number *n* of cells is indicated within each bar. **G** Confocal fluorescence images of CFP-Orai1 WT and YFP-STIM1 L251S ± F394D (CFP in green, YFP in red) as well as an overlay (Merge in yellow) for visual comparison. Images were captured in the periphery of the cells. Length of scale bars corresponds to 5 µm. Data represent mean values ± SEM. HEK293 cells were used for all experiments. Student’s two-tailed *t* test was employed for statistical analyses with differences considered statistically significant at *p* < 0.05. Asterisks (*) indicate significant difference. Color code: WT (black), Δα2 (red), F394A (dark cyan), F394D (blue), F394H (orange)

An intramolecular clamp established by CC1 and CC3 in the STIM1 C-terminus maintains the quiescent state of STIM1 [16, 18]. Store-operated activation of STIM1 involves the release of this inhibitory interaction leading to the exposure of CAD/SOAR that supports STIM1 homomerization and coupling to Orai1. The single point mutation L251S in CC1 is known to induce this conformational switch from the inactive to the active state [16]. This can be impressively shown by the Orai activating small fragment (OASF) conformational sensor construct, composed of the cytosolic OASF fragment (STIM1 233–474) (Fig. 2A) labelled at both the N- and C-terminus with YFP- and -CFP, respectively (YFP-OASF-CFP, Fig. 3A). Here, we investigated whether the deletion of α2 or mutations of F394 (F394H, F394D, F394A), that drastically impaired homomerization of full-length STIM1 and Orai1 activation, affect the ability of STIM1 OASF to release the inhibitory clamp. Deletion of α2 within or the introduction of F394H, F394D or F394A into YFP-OASF-CFP revealed for all the sensor mutants robust FRET comparable to the WT form indicating the closed state (Fig. 3B, C). Thus, these mutations do not alter the quiescent state of the cytosolic YFP-OASF-CFP construct. This is especially surprising for the F394A substitution which induced constitutive activity of full-length STIM1 (Fig. 2B). The additional introduction of L251S in the YFP-OASF-CFP variants resulted in a decrease in FRET to a comparable extent, suggesting that all mutants switch into an extended conformation (Fig. 3B, C), even though the deletion of α2 or the F394H/D substitutions within full-length STIM1 led to impaired function (Fig. 1B, Fig. 2E).

We previously discovered that full-length STIM1 containing the L251S mutation exhibited constitutive puncta formation, coupling to and activation of Orai1 channels [16] due to the release of the inhibitory clamp. Thus, we examined in the following whether the additional L251S substitution in full-length STIM1 Δα2 or the STIM1 F394X (X = A, D, H) mutants restores any step of the STIM1/Orai1 activation cascade. STIM1 L251S Δα2 exhibited puncta formation, co-localized with Orai1 subsequent to store-depletion and induced activation of constitutive Orai1 currents, although, only in ~ 30% of the experiments (Fig. 3D–F; Supp. Figure 4D). In S1/S2 dKO HEK 293 cells, STIM1 L251S Δα2 also led to constitutive currents, however, without increasing in size as observed in normal HEK293 cells (Fig. 3D), suggesting that endogenous STIM1 supports the activity of STIM1 L251S Δα2 (Supp. Figure 3B). STIM1 L251S F394H showed constitutive puncta formation, but weak co-localization with Orai1 upon store-depletion and led to tiny store-operated Orai1 current activation, however only in 60% of the cells (Fig. 3D–F; Supp. Figure 4D). STIM1 L251S F394D exhibited no puncta formation, was unable to co-localize with Orai1 upon store-depletion and interfered with the stimulation of Orai1 currents (Fig. 3D–G). STIM1 L251S F394A led to puncta formation, constitutive co-localization with Orai1 as well as current activation independent of store-depletion (Fig. 3D–F; Supp. Figure 4D).

Summarizing, in the OASF, inhibitory mutations in α2 do not interfere with the conformational switch from the quiescent to the active state induced by L251S, as shown with the cytosolic YFP-OASF-CFP conformational sensor. In the context of ER-anchored full-length STIM1, however, the release of the inhibitory clamp via the L251S mutation is not sufficient to restore co-localization of STIM1 L251S F394D with and activation of Orai1.

### In the OASF, F394 controls communication with Orai1, but not homomerization

**Fig. 4:**
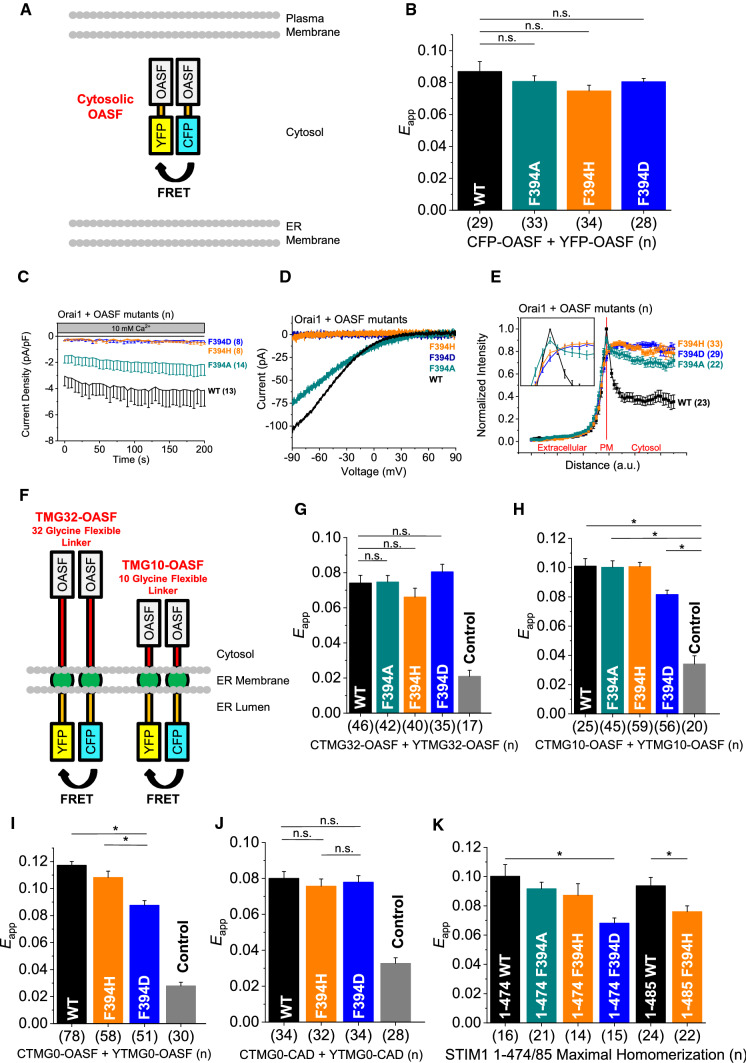
F394 controls oligomerization when OASF is tightly anchored in the membrane. **A** Scheme of cytosolic OASF homomerization. Upon interaction of two OASF molecules, the fluorophores CFP and YFP come into close proximity, allowing FRET to occur. **B** Intermolecular homomerization FRET (*E*_app_) measurements of cytosolic CFP-OASF + YFP-OASF fragments using the following constructs: OASF WT, F394A, F394D, and F394H. **C** Time course of whole cell inward currents at − 74 mV of HEK293 cells co-expressing Orai1 WT together with cytosolic OASF constructs using the same mutants used in (**B**). **D** Corresponding I/V relationships of maximum currents shown in (**C**). **E** Normalized fluorescence intensity plots of cytosolic OASF constructs using the same mutants as in (**B**) co-expressed with Orai1 showing localization in regions close to the plasma membrane. Inset showing normalized intensity at and in the surrounding of the plasma membrane (PM). **F** Cartoon representation of the FIRE system. The N-terminal fluorophores CFP and YFP within the ER lumen are bound to the STIM1 TM domain. On the cytosolic C-terminal side, a flexible linker with a length of either 32 or 10 glycines (red bars) is used to attach the STIM1 OASF domain to the ER membrane. **G** Intermolecular FIRE (*E*_app_) homomerization measurements of ER membrane-bound CFP-TMG32-OASF + YFP-TMG32-OASF fragments (C/YTMG32-OASF) containing a flexible cytosolic linker consisting of 32 glycines using the same mutations as in (**B**). For the control, CTMG32-OASF was co-expressed with a YTMG32 construct lacking OASF (*cf.* Supp. Figure 14B). Control *E*_app_ is significantly smaller than all tested OASF interactions (not indicated). **H**–**J** Same as in (**G**) but for ER membrane-bound C/YTMG10-OASF (**H**), C/YTMG0-OASF (**I**) or C/YTMG0-CAD (**J**) fragments containing a shorter flexible cytosolic linker consisting of only 10 glycines (**H**) or no linker (**I**, **J**). Control *E*_app_ is significantly smaller than all tested OASF (**H**, **I**) or CAD (**J**) interactions (not indicated). **K** Intermolecular homomerization FRET (*E*_app_) measurements of cytosolic CFP-STIM1 1–474 + YFP- STIM1 1–474 fragments using the following constructs: STIM1 1–474 (wild-type), F394A, F394H, and F394D and CFP-STIM1 1–485 + YFP-STIM1 1–485 fragments using the following constructs: STIM1 1–485 (wild-type) and STIM1 1–485 F394H. Data represent mean values ± SEM. HEK293 cells were used for all experiments. Student’s two-tailed *t* test was employed for statistical analyses with differences considered statistically significant at *p* < 0.05. Asterisks (*) indicate significant difference. Color code: WT (black), F394A (dark cyan), F394D (blue), F394H (orange), Control (gray)

To further corroborate the divergent behavior of the F394X mutations in the OASF sensor compared to full-length STIM1 in the presence of the L251S mutation (Table 1), we performed additional experiments on the OASF domain level (Fig. 4A). Interestingly and in contrast to full-length STIM1 (Fig. 2; Table 1), the corresponding cytosolic OASF mutants containing a F394X (X = A, D, H) substitution exhibited homomerization comparable to OASF WT in FRET experiments (Fig. 4B). Investigation of OASF mutant mediated Orai1 activation revealed constitutive activity for cytosolic OASF F394A in the presence of Orai1, but with a 50% reduction in current size compared to WT (Fig. 4 C, D). OASF F394D and F394H were insufficient to activate Orai1 currents (Fig. 4C, D). To probe co-localization between soluble OASF constructs and Orai1, we carried out line scan experiments [16, 27] to measure the distribution of OASF confocal fluorescence intensity from the extracellular space across the plasma membrane into the cytosol. For OASF WT, a distinct peak is visible due to its binding to and activation of Orai1 in the plasma membrane (Fig. 4E). Consistent with our patch clamp results, OASF F394A was the only mutant which was partly able to mimic the WT behavior, as visible by a distinct, but less sharp peak in the line scan (Fig. 4E). Moreover, OASF F394D and F394H both showed a gradual increase in fluorescence intensity without any distinct peak that is indicative of disrupted binding to Orai1 (Fig. 4E) in line with the functional results (Fig. 4C, D). In support, additional FRET experiments revealed comparable interaction of YFP-OASF and YFP-OASF F394A with CFP-Orai1, but significantly reduced coupling of YFP-OASF F394D and YFP-OASF F394H with CFP-Orai1 (Supp. Figure 6A).

Overall, mutations of F394 in α2 of the cytosolic OASF fragment interfere either partially (F394A) or fully (F394D/H) with activation of and co-localization with Orai1, but leave OASF homomerization unaffected. Unaffected homomerization of OASF F394X mutants is in line with unaffected switch to the extended conformation of the OASF sensor upon L251S substitution (Fig. 3B). However, these findings are in sharp contrast to the strongly reduced or abolished homomerization of analogue full length STIM1 mutants (Table 1). This may indicate that α2 in the OASF fragment predominantly controls the coupling to Orai1. Contrary, in full-length STIM1, α2 likely governs in particular homomerization and, only in a step downstream, also coupling to Orai1.

### Attachment of OASF F394D to the membrane decreases homomerization

To determine whether the anchoring of STIM1 in the ER membrane is responsible for the differences in homomerization between STIM1 and OASF α2 mutants, respectively, we employed our FRET-derived interaction in a restricted environment (FIRE) method [18]. For that we initially linked the OASF fragments to the ER membrane via a flexible 32-glycine linker (TMG-32-OASF) to mimic native ER-targeted two-dimensional localization rather than using cytosolic expression (Fig. 4F). All TMG-32-OASF mutants carrying a F394X (X = A, D, H) mutation displayed homomerization comparable to the soluble OASF fragments (Fig. 4B, G). Concerning the co-localization with and activation of Orai1, the behavior of the ER membrane-linked TMG-32-OASF mutants largely corresponded to their soluble OASF counterparts. (Fig. 4C–E; Supp. Figure 6B, C).

Next, we engineered an OASF construct linked via a 10-glycine linker (TMG-10-OASF) or directly to the ER membrane (TMG-0-OASF), in order to reduce the flexibility of the OASF fragment and increase the proximity to the ER membrane (Fig. 4F). Indeed, TMG-10-OASF F394D and TMG-0-OASF F394D exhibited significantly reduced homomerization, compared to corresponding OASF WT (TMG-10-OASF WT, TMG-0-OASF WT) and OASF-F394A (TMG-10-OASF-F394A) TMG fragments (Fig. 4H, I). Interestingly, also TMG-10- OASF F394H and TMG-0-OASF F394H showed robust homomerization FRET, suggesting that it still possesses the ability to establish homomerization (Fig. 4H, I).

Additionally, we designed a CAD construct linked directly to the ER membrane (TMG-0-CAD). Remarkably, we observed that TMG-0-CAD F394D and TMG-0-CAD F394H exhibited comparable homomerization like TMG-0-CAD WT (Fig. 4J).

Moreover, we engineered a STIM1 truncation mutant, STIM1 1–474, representing physiological conditions with the OASF fragment directly fused via CC1 to the STIM1 TM domain. In accord with the degree of homomerization of the TMG-10-/0-OASF fragments, STIM1 1–474 F394D exhibited abolished homomerization compared to STIM1 1–474 and STIM1 1–474 F394A. Unexpectedly, STIM1 1–474 F394H showed store dependent homomerization comparable to WT STIM1 1–474 (Fig. 4K, Supp. Figure 6D). Hence, we further investigated the effect of the CRAC modulatory domain (CMD; aa 474–485) on the homomerization of the STIM1 truncation mutant (STIM1 1–485). Indeed, while STIM1 1–485 F394H exhibited abolished homomerization, that of STIM1 1–485 remained preserved (Fig. 4K, Supp. Figure 6E). This suggests an additional modulatory role of CMD on the apex region.

These observations suggest that mutations in the α2 region can affect homomerization only in the context of full-length STIM1 or when OASF is anchored to the ER membrane. In contrast, these LoF mutations in the apex do not impact homomerization when incorporated into a soluble STIM1 C-terminal fragment or when inserted into CAD that is tethered to the ER membrane.

### Simulated F394 substitutions enhance the CC1α1-CAD/SOAR clamp and interaction of the apex with the ER membrane

**Fig. 5:**
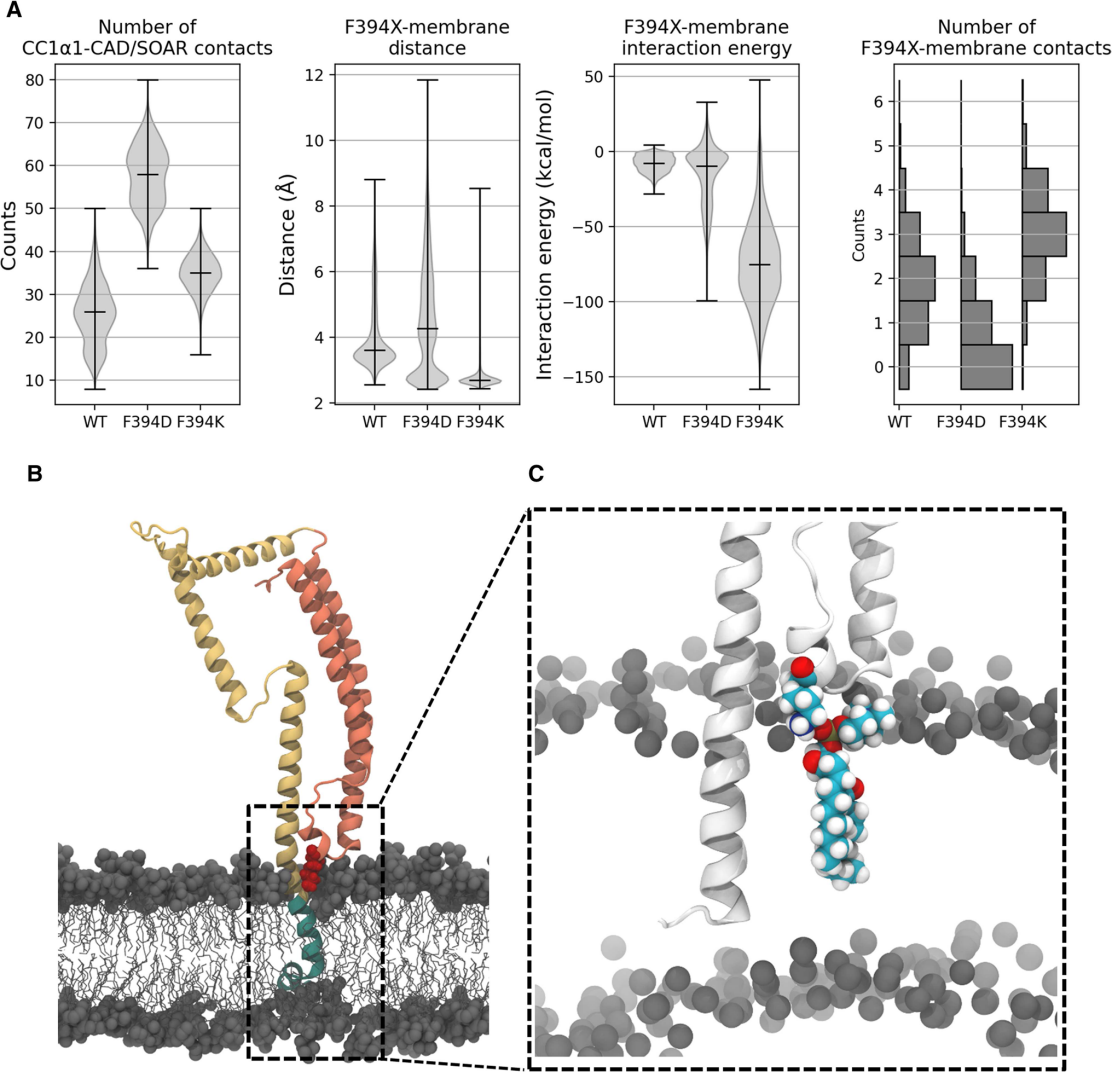
F394 is located close to the ER membrane in molecular dynamics simulations. **A** Number of CC1α1-CAD/SOAR contacts, distances between closest non-hydrogen atoms of the membrane and X394, X394-membrane interaction energy and number of contacts between X394 and the membrane (X = F,D,K). Medians and extrema are denoted by a black line. **B** Side-view of the F394K model. The TM, CC1 and CAD/SOAR domains are coloured in green, yellow and orange, respectively. K394 is shown in red, forming numerous contacts with lipid headgroups (grey). **C** Zoom-in onto the CAD/SOAR apex. K394 and one DDPC molecule, which forms near-permanent contacts with K394, are shown in space-filling representation

To gain further insight into the role of the α2 region, specifically of the position F394 in ER-bound STIM1, we performed molecular dynamics (MD) simulations. We designed a model of STIM1 which comprises residues 214–443, including the TM, CC1 and CAD/SOAR domains (Fig. 5B; Supp. Figure 7). The model was built based on known interaction sites between CC1 and CAD/SOAR [37], by docking the CAD/SOAR crystal structure (PDB ID 3TEQ, [28]) to α1 of the CC1 NMR model (PDB ID 6YEL, [19]) and fitting the rest of the structure to the docked conformation (see Methods and Supp. Figure 1, Supp. Figure 7 for details).

According to our model, F394 occupies a rather exposed position at the apex of the CAD/SOAR domain. Due to the position of the CAD/SOAR apex and our results shown in Fig. 4, we suspected that interactions with headgroups of the ER membrane phospholipids could prove pivotal for a charged substitution like F394D/K. We thus embedded our model in a membrane composed of phosphatidylcholine (DDPC) and phosphatidylethanolamine (DLPE) with a ratio of 2:1 [44] (Supp. Figure 8). In the resultant structure, residue 394 was located in the immediate vicinity of the ER membrane (at a distance of only a few Å, Fig. 5, Supplementary Movie).

We found that compared to the WT, F394D showed a distinctively increased number of contacts between CC1α1 and CAD/SOAR, indicating a strengthening of the STIM1 inhibitory clamp (Fig. 5A). This enhancement of contacts occurred not only in the immediate vicinity of the mutated position 394 but along the entire length of the CC1α1-CAD/SOAR interface (Supp. Figure 9). While electrostatic interaction between CC1α1 and CAD/SOAR remained, on average, largely unaffected by the F394D mutation, the number of hydrophobic CC1α1-CAD/SOAR contacts was significantly increased (Supp. Figure 10).

In our simulation of the F394K mutant we also observed an enhancement in contacts along the CC1α1-CAD/SOAR interface, although not as pronounced as for F394D (Supp. Figure 9, 10A). Moreover, we found that compared to the WT, F394K attached much more strongly and frequently to phospholipid headgroups. As a positively charged amino acid, K394 was strongly attracted to negatively charged phosphate groups in membrane phospholipids (Fig. 5A, C). Compared to the WT, F394K increased the mean number of contacts between the membrane and residue 394 by approximately 50%. K394 was bound to one specific DDPC molecule throughout 71% of our simulation, while in the WT the longest-lasting contact with any single lipid persisted for 31% of the simulation time.

The above-mentioned simulations were performed at physiological ion concentration. In addition, we conducted MD simulations of further STIM1 variants (F394X, X = A, H, F, D, K) under neutralizing ion conditions (see Methods) to clearly accentuate potential electrostatic interactions between residue 394 and polar headgroups of phospholipids of the ER membrane. It is worth noting that modelling the exact ion concentration very close to the membrane would require more extensive simulations of the crowded membrane surface. At low ion concentrations, F394K, F394D and positively charged F394H^+^ bind more closely to the membrane than the WT and hydrophobic F394A (Supp. Figure 11). As an additional negative control, we performed a simulation of F394D embedded in a membrane consisting entirely of negatively charged phosphatidylserine (PS). As expected, the CAD/SOAR apex was repelled from the membrane due to electrostatic repulsion (Supp. Figure 12).

Electrostatic D/K394-ER membrane interactions affixed the CAD/SOAR domain to the membrane and, in the case of F394D, even allowed hydrophobic portions of the CAD/SOAR apex (L390, F391) to partly partition into the membrane (Supp. Figures 11, 13). We found uncharged F394H^0^ to be an intermediate case insofar as it formed more contacts with lipid phosphate groups and interacted more strongly with the membrane than the WT, but less than e.g., F394K. F394A behaved much like the WT, with both remaining separated from the lipid phase as shown by similar distances to the membrane and comparable membrane interaction energy (Supp. Figure 11). Both F394 and A394 formed few contacts with the membrane and attached rather loosely.

Neutralizing ion conditions seemed to decrease D394 solubility and thus benefited D394-membrane binding. In contrast to low ion concentrations, physiological bulk concentrations favor reversible detachment of D394 from the membrane and strengthen hydrophobic CC1α1-CAD/SOAR interactions in the F394D mutant. It seems that the cause of stabilization of the STIM1 quiescent state in this mutant shifts from enthalpic contributions (the electrostatic CAD/SOAR-membrane interaction) at low ionic strength to predominantly entropic contributions (the strengthening of the hydrophobic CC1α1-CAD/SOAR interface) at physiological bulk ion concentrations. By contrast, in the case of F394K the enthalpic contributions dominate at both ion concentrations investigated.

In summary, structural modeling and MD simulation of a TM-CC1-SOAR fragment suggest close proximity of the CAD/SOAR apex to the ER membrane. Positively charged F394K and F394H^+^ were electrostatically attracted to polar headgroups of ER membrane phospholipids, whereas F394 and F394A attached more weakly to the membrane. The F394D mutation enhanced the STIM1 inhibitory clamp, as indicated by an increase of CC1α1-CAD/SOAR contacts, especially between hydrophobic residues. In analogy to electrostatic interactions of F394K and F394H^+^ with negative charges at the lipid headgroups, negatively charged D394 strongly binds to NH^3+^ moieties in phospholipid headgroups in our simulations performed at lower ion concentration. All in all, either mechanism (binding of charged residues to the membrane or a reinforcement of the CC1α1-CAD/SOAR binding) may contribute to the impaired STIM1 oligomerization observed in our experiments.

### F394 in OASF α2 is crucial for interaction with Orai1 N- and C-terminus

We discovered that defect STIM1 α2 mutants are predominantly impaired in homomerization due to tight attachment of the mutated α2 region to the ER membrane (Fig. 1E, F, Fig. 2D, G). Contrary, dysfunctional OASF α2 mutants retained homomerization, but still affected coupling to and activation of Orai1 (Fig. 4B–E). Despite constitutive homomerization and coupling to Orai1, STIM1 F394A and OASF F394A exhibited only half-maximal Orai1 current activation compared to those obtained with STIM1 WT (Fig. 2B, H, Fig. 4B–E). It has been suggested previously that F394 potentially interplays with Orai1 N-terminus or the Orai1 hinge plate, which represents a hydrophobic interaction site of TM3 and TM4 close to the bent region connecting TM4 and C-terminus [55, 64]. Thus, we investigated whether α2 mutations interfere with the coupling to one of the three cytosolic regions: N-terminus, loop2 and C-terminus, of Orai1 (Supp. Figure 14A). It is well-known that Orai1 C-terminus represents the main coupling site for STIM1. Nevertheless, there is clear evidence that STIM1 α3 relays the gating signal to the pore via direct interaction with a region in loop2 (aa 160–170) close to TM3 [30]. Moreover, we reported that the communication of STIM1 and Orai1 requires an intact N-terminus to fully restore activation and biophysical properties of CRAC channels [32, 65–67]. Additionally, we recently provided indications that the loop2 functions as a bridge for transferring the Orai1 activation signal induced via STIM1 coupling from the Orai1 C-terminus to its N-terminus [67, 68]. However, since Orai1 C-terminus is an indispensable requisite for STIM1 coupling to and subsequent activation of Orai1, it is not trivial to determine within full-length Orai1 whether STIM1 couples to the other two cytosolic regions, N-terminus and loop2, directly. Thus, we used the FIRE system [18] to investigate the effect of α2 mutations in OASF on the coupling to cytosolic fragments of Orai1 (Supp. Figure 14B). We discovered robust coupling of the STIM1 OASF fragment [TMG-OASF (aa 233–474)] with all three cytosolic Orai1 fragments, N-terminus [TMG32-N-terminus (aa 1–90)], loop2 [TMG32-loop2 (aa 137–184)] and C-terminus [TMG32-C-terminus (aa 255–301)] (Supp. Figure 14C–E). With respect to the N-terminus, all F394X (X = A, H, D) mutations led to significantly reduced coupling of the respective OASF fragment compared to WT (Supp. Figure 14C). None of the F394X mutations interfered with the coupling of OASF and the loop2 segments (Supp. Figure 14D). Interestingly, only F394D, but not F394A and F394H, significantly reduced FRET of the OASF mutant with Orai1 C-terminus (Supp. Figure 14E).

In summary, we demonstrated that mutation of F394 in the STIM1 C-terminal α2 region can disturb the interaction with the Orai1 N-terminus and the Orai1 C-terminus. While all F394 mutations reduce the interaction of OASF with the Orai1 N-terminus, only F394D impairs the coupling of OASF with the Orai1 C-terminus. The α2 region in OASF does not seem to be required for a communication with the loop2 of Orai1.

## Discussion

**Fig. 6:**
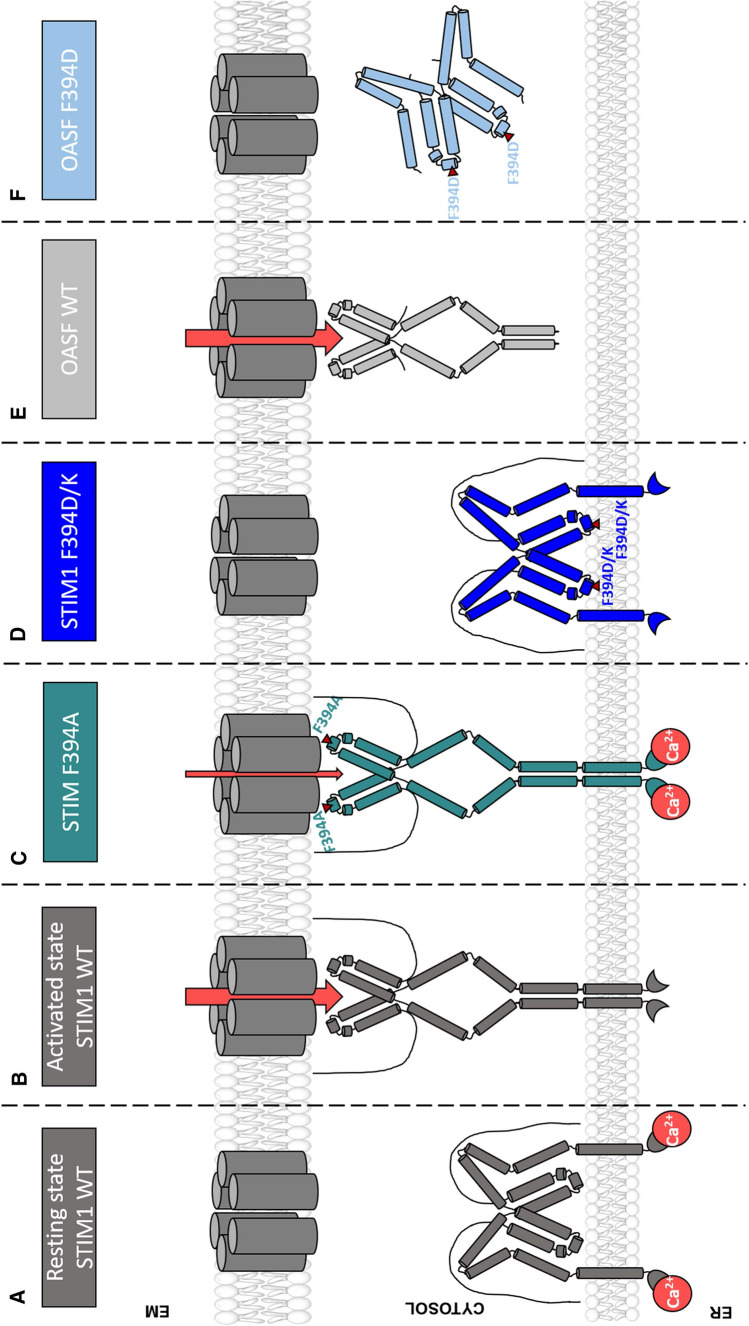
Multiple effects of F394 mutations in the α2 region on diverse steps in the STIM1/Orai1 activation cascade. **A** Under resting conditions in the cell, the STIM1 C-terminus is kept in a quiescent state via interactions within the inhibitory clamp. The SOAR apex is located close to the ER membrane. **B** Upon Ca^2+^ store depletion of the endoplasmic reticulum (ER), STIM1 loses its bound Ca^2+^ and undergoes a conformational switch into an extended state capable of binding to Orai1 channels. Following this, the channels become activated and Ca^2+^ can enter the cell. **C **STIM1 F394A is constitutively active, but activates Orai1 channels to a reduced extent. **D** For STIM1 F394D/K, the conformational switch upon store depletion is prevented by altered formation of the CC1α1-SOAR/CAD interface and/or the formation of electrostatic interactions to the ER membrane. **E** The soluble OASF fragment (aa 233–474) induces constitutive activation of Orai1. **F** The OASF F394D mutant is able to oligomerize as it is not attached to the ER membrane, but is still impaired in coupling to and activation of Orai1

In this study, we showed that distinct amino acid substitutions of F394, within the small alpha helical domain α2 in the CAD/SOAR region of STIM1 can lead to multifaceted effects in the STIM1-Orai1 activation process (Fig. 6). The most severe effects were observed upon substitutions to residues with charged, hydrophilic side chains, which lead to loss of STIM1 homomerization likely due to an altered CC1α1-SOAR/CAD interface and/or electrostatic interaction with phospholipid headgroups in the membrane. Our mutational studies provide for the first time evidence that the STIM1 apex is oriented towards and close to the ER membrane.

It has been reported [55] that a specific amino acid within STIM1 α2 (F394) is involved in the activation of Orai1 channels. Therefore, it was proposed that the α2 region acts as a putative gating/coupling site. Substitution by a histidine at this position (F394H) resulted in impaired STIM1 cluster formation and Ca^2+^ influx [55]. Indeed, our data support the profound functional impact of this mutation. Nevertheless, we observed Orai1 current activation upon store depletion in approximately one third of the experiments with STIM1 F394H, though with significantly reduced maximum current levels, together with moderate homomerization and co-localization with Orai1. The effect was even more severe in STIM1 F394D/K mutants, which completely abolished homomerization, co-localization with and activation of Orai1 (Fig. 6D). These results were further supported by the application of 2-APB, where homomerization of STIM1 F394H, but not STIM1 F394D, was fully restored, and puncta formation, as well as co-localization with Orai1 reached levels comparable to STIM1 WT. Deletion of the entire α2 domain in STIM1 resulted in Orai1 currents of reduced size in only about half of our experiments, whereas co-localization with Orai1 was much less affected than homomerization. The L251S mutation, known as a GoF mutation due to the release of the inhibitory CC1-CC3 clamp of STIM1, was ineffective for restoring puncta formation and activation of Orai1 of severely dysfunctional STIM1 α2 mutants. Furthermore, a serine substitution (STIM1 F394S) led to partial interference with STIM1 homomerization and STIM1-Orai1 coupling. Altogether, these inhibitory STIM1 α2 mutations predominantly interfere with STIM1 homomerization, while disruption of coupling to and activation of Orai1 occur only in a downstream step within the STIM1/Orai1 activation cascade.

Interestingly, STIM1 F394A showed constitutive homomerization in accordance with constitutive coupling to and activation of Orai1. Despite the constitutive activity of STIM1 F394A, it is worth noting that maximal currents reached with Orai1 were reduced by 50% compared with WT STIM1 (Fig. 6C). This alanine substitution in the α2 region is able to destabilize the quiescent state of STIM1 and thereby facilitate the activation of Orai1, albeit to a reduced extent.

The inhibitory effects of the diverse STIM1 α2 mutants in the STIM1/Orai1 activation cascade are supported by our data showing that the constitutively active Orai1 P245L mutant only in part facilitated their store-operated coupling and activation. Moreover, authentic CRAC channel hallmarks indicative of proper STIM1-Orai1 interplay [60] were impaired for most STIM1 α2 mutant mediated Orai1 P245L currents.

In contrast to all defect full-length STIM1 α2 mutants, analogous STIM1 OASF α2 mutants exhibited homomerization comparable to STIM1 OASF WT. Linkage of OASF α2 mutants to the ER membrane via a 32-glycine linker led to comparable homomerization propensity, likely due to sufficient flexibility comparable to cytosolic OASF fragments. Only OASF mutant fragments anchored closer to the membrane, as achieved with a 10-glycine linker or direct attachment (without linker) and STIM1 mutants truncated after OASF (STIM1 1–474, STIM1 1–485), showed a reduction of homomerization for the F394D substitution comparable to analogue full-length STIM1 mutants. In contrast, CAD fragments directly tethered to the ER membrane exhibited no alteration in homomerization when containing the F394D mutation. Collectively, these findings suggest that anchoring of OASF, but not CAD, near to the ER membrane is responsible for impaired homomerization of the hampered STIM1 α2 mutants (Fig. 6D–F). Indeed, MD simulations with a STIM1-TM-CC1-SOAR fragment revealed that the CAD/SOAR apex is in close proximity to the ER membrane. Site-directed mutagenesis at position F394 led to alterations in the distance between the apex and the ER membrane together with a shift in the CC1α1-CAD/SOAR binding interface. In particular, we determined for F394D a drastic alteration in the hydrophobic interaction along the CC1α1-CAD/SOAR interaction interface. F394K led to strongly enhanced interaction with the phospholipid headgroups in the ER membrane together with a slight shift in the CAD/SOAR binding interface. Interestingly, we observed under neutralized simulation conditions strong electrostatic interactions between the charged side chains of F394D or F394K and headgroups of phospholipids of the ER membrane. This may explain the contrasting homomerization results of cytosolic OASF F394D, TMG-32-OASF F394D and TMG-0-CAD F394D compared with full length STIM1 F394D, STIM1 1–474/485 F394D as well as TMG-10-/TMG-0-OASF F394D. When OASF is floating around in the cytosol, the CAD/SOAR apex is likely not close enough for interaction with the ER membrane, while in full length STIM1 it comes in closer proximity to the ER membrane. Based on structural [28] studies it can be assumed that in contrast to TMG-0-OASF, in TMG-0-CAD the apex is far apart from the ER-membrane, thus, unable to form interactions with the lipid headgroups therein. The loss of homomerization of STIM1 F394D suggests that the inhibitory clamp formed by CC1 and CC3 within STIM1 C-terminus cannot be released due to tighter attachment of CC1α1 to SOAR/CAD region and possibly additional electrostatic interactions of the CAD/SOAR apex with phospholipid headgroups in the ER membrane. Supportively, we demonstrated that constitutive puncta formation of the STIM1 L251S mutant, which occurs due to the release of the inhibitory clamp, was impaired for STIM1 L251S F394D.

The histidine at position 394 (F394H) occupies an intermediate state, as indicated via the partial activity of STIM1 F394H regarding its homomerization, coupling to and activation of Orai1. The behavior can be rationalized with the approximately 50% protonation chance of the histidine side chain at physiological pH. Consistent with this, MD simulations have shown that the protonated histidine interacts more strongly with the ER membrane than the neutral histidine. In the context of constitutively active STIM1 F394A, MD simulations revealed a similar distance of α2 to the membrane for this mutant compared to STIM1 WT. Therefore, we mechanistically suggest that the GoF of STIM1 F394A is more likely to occur due to altered hydrophobic interactions compared to the larger and more hydrophobic amino acids at this position, thus counteracting the quiescent state of STIM1.

Moreover, our results indicate for the first time the orientation of CAD/SOAR with respect to the entire STIM1 protein of a resting cell. Under resting conditions, STIM1 is in a tightly packed conformation due to intramolecular STIM1 CC1-CC3 interaction [16, 18, 69]. However, whether the CAD/SOAR apex is oriented towards the ER membrane or away from the ER membrane has been controversial [70]. Our MD simulations and functional data provide novel evidence for the close proximity between the ER membrane and the CAD/SOAR apex. It is worth noting that during the preparation of this manuscript, a similar STIM1 model was published by van Dorp et al. [71]. Their model was created by docking CC1α1 to CAD/SOAR using the Rosetta webserver with distance restraints based on single-molecule FRET measurements. In close analogy, we docked the same fragments using HADDOCK with restraints based on functional and FRET data by Ma et al. [37]. Both methods lead to similar results concerning the CC1α1-CAD/SOAR docking interface (Supp. Figure 7). In the model proposed by van Dorp et al. [71], CAD/SOAR docks to CC1α1 such that the CAD/SOAR apex is closer to the luminal membrane leaflet and the CAD/SOAR apex is apposed to the STIM1 transmembrane helix, suggesting that CAD/SOAR would deeply penetrate the ER membrane. The similarity between those two models reinforces our conclusion that in the quiescent STIM1, the CC1α1-CAD/SOAR clamp is formed such that F394 and its substitutions are located close to and electrostatically interact with the ER membrane. The analysis of the functional relevance of the STIM1 apex in maintaining the STIM1 quiescent state via interactions with the ER membrane is a promising perspective for future research. Nevertheless, we assume that the LoF induced by F394D or F394K can be explained by a combined effect of altered interaction in the CC1α1-CAD/SOAR binding interface and the tight attachment of the apex to the ER membrane.

Although OASF α2 mutants exhibit intact homomerization, they fail to couple to and activate Orai1. Even OASF F394A exhibited only partial constitutive activation compared to OASF WT. Analogously, STIM1 F394A showed reduced maximal current activation despite constitutive homomerization and Orai1 activation. Altogether, these results highlight that the α2 region also controls coupling to and activation of Orai1. Indeed, our OASF-FIRE approach revealed that all F394X (X = A, D, H) substitutions affected coupling to the Orai1 N-terminus. Additionally, the most severe mutation, F394D, resulted in a strongly decreased interaction with the Orai1 C-terminus, likely abrogating Orai1 interaction with OASF F394D.

In summary, our set of STIM1 α2 mutants reveal that the α2 region occupies a more complex role in CRAC channel activation than originally thought. Single point mutation of F394 in the α2 region influences a range of steps within the STIM1/Orai1 activation machinery, including the transition from the closed to the open state of STIM1, homomerization, co-localization with and activation of Orai1. Herewith, we provide compelling evidence that sufficient STIM1 homomerization and co-clustering with Orai1, both of which are indispensable for Orai1 activation, can be drastically impaired by the properties of the STIM1 CAD/SOAR apex, most notably by residue 394.

### Supplementary Information

Below is the link to the electronic supplementary material.

Supplementary file1 (PDF 12343 kb)

Supplementary file2 (MP4 202769 kb)

## Data Availability

Data supporting the findings of this manuscript are available from the corresponding authors upon reasonable request.
