# Supplementary material for: Defects in the STIM1 SOARα2 domain affect multiple steps in the CRAC channel activation cascade
Source: Cell Mol Life Sci. 2021 Sep 8;78(19-20):6645–67. doi: 10.1007/s00018-021-03933-4 (PMC8558294; doi:10.1007/s00018-021-03933-4)
Supplement: Supplementary file 1 — Supplementary file1 (PDF 12343 kb) [file 18_2021_3933_MOESM1_ESM.pdf]

Supplementary Material

Supplementary Table 1:

Nr	Score	Extent	SD	CC1α1-CC3 orientation	RMSD	SD	VdW	SD	Elec	SD	Restr	SD	BSA	SD	Desolv	SD
7	-79,3	5	18,8	antiparallel	0,8	0,5	-33,7	9,0	-216,4	37,4	13,5	7,6	1593,7	250,5	-3,7	6,4
1	-72,7	18	7,5	antiparallel	1,2	0,7	-38,1	3,5	-150,2	11,6	24,0	13,3	1341,3	99,3	-7,0	5,8
2	-68,5	9	5,7	parallel	0,8	0,5	-40,5	4,8	-86,0	17,3	18,8	23,5	1512,4	74,1	-12,7	6,2
**4**	**-60,9**	**7**	**8,3**	**parallel**	**1,6**	**1,0**	**-26,1**	**5,8**	**-133,4**	**30,6**	**2,6**	**1,2**	**1007,0**	**104,9**	**-8,4**	**8,4**
6	-55,0	6	6,8	antiparallel	1,6	1,0	-30,3	6,5	-133,2	37,7	22,3	12,0	991,8	129,0	-0,3	3,6
11	-51,7	4	12,3	antiparallel	0,9	0,6	-27,7	6,4	-153,0	16,5	37,4	20,0	1106,1	103,3	2,9	4,0
3	-50,3	8	5,4	antiparallel	0,9	0,6	-33,1	5,0	-107,3	12,0	29,0	24,7	1085,1	86,3	1,3	3,0
9	-40,2	4	9,3	parallel	1,0	0,7	-29,4	1,7	-35,4	10,5	2,8	0,6	1057,5	155,6	-4,1	6,7
5	-29,0	6	2,4	crossways	2,3	1,3	-20,7	4,3	-122,1	32,0	20,5	17,9	906,4	43,8	14,0	6,0
10	-28,9	4	11,4	parallel	1,1	0,7	-29,7	8,7	-40,7	22,6	42,8	10,1	1071,0	210,7	4,6	6,8

Docking clusters

Overview of the results obtained from the HADDOCK docking simulation. “Extent” denotes the number of structures contained in the respective cluster. RMSD is calculated with respect to the overall lowest-energy structure (in Å). “VdW” denotes van der Waals energy. “Elec“ denotes electrostatic energy. “Restr” refers to restraints violation energy. “BSA” denotes buried surface area. “Desolv” denotes desolvation energy. “SD” denotes the standard deviation.

Supplementary Figure 1:

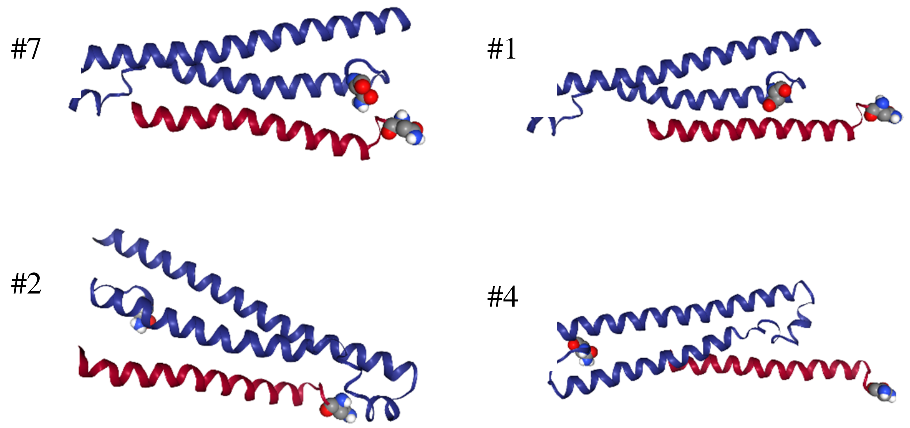

The four top-scoring HADDOCK cluster clusters (#7, 1, 2, 4) are shown. The CAD/SOAR domain is displayed in blue and CC1α1 in red. For reference, the CC1α1 N-terminus and the CAD/SOAR C-terminus are shown in space-filling representation. In cluster #7 and #1, CAD/SOAR apex is oriented away from the ER membrane and would lead to very unfavourable placement of CC1α2/3. When taking into account the presence of the ER membrane, cluster #2 would lead to steric clashes. Of the top-scoring clusters, cluster #4 best fits the expected STIM1 quiescent conformation. It was thus chosen as a starting point for our molecular dynamics simulations.

Supplementary Figure 2 A:

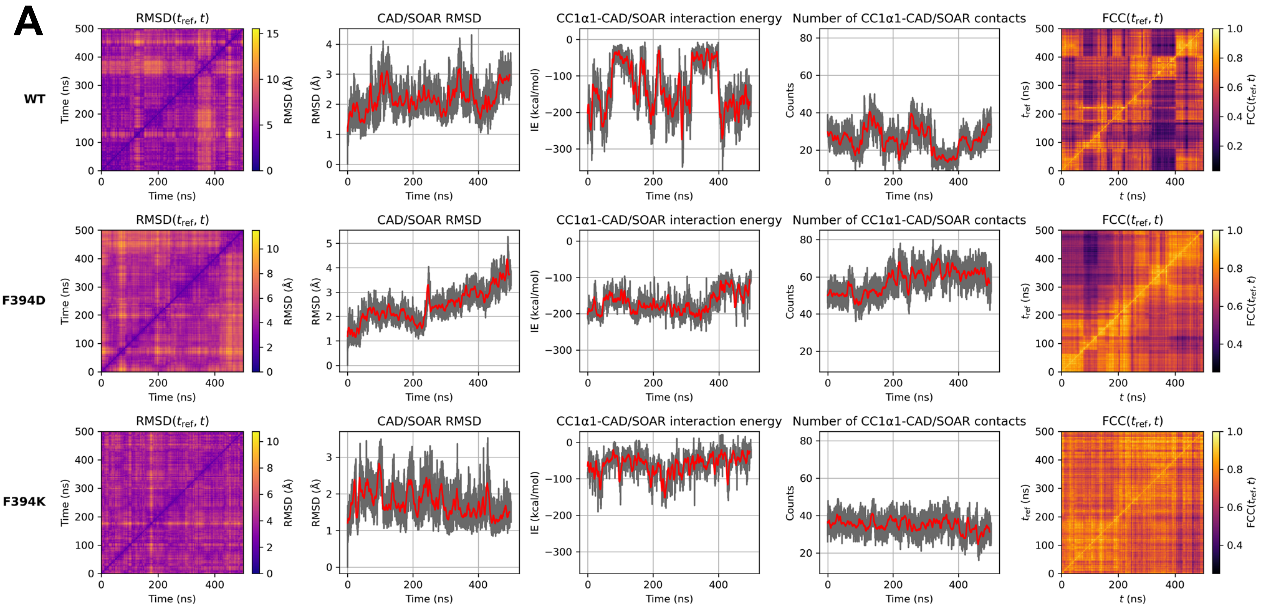

Structural stability

Structural stability for the final 500 ns of simulations of the WT, F394D and F394K performed at 0.15 M KCl. For each run, CA-atom based all-to-all RMSD(t, t_{ref}), RMSD$(t, 0)$ for the CAD/SOAR domain, CC1α1-CAD/SOAR interaction energy (IE), the number of CC1α1-CAD/SOAR contacts and the all-to-all fraction of common contacts (FCC) map FCC(t, t_{ref}) is shown. The contact number was calculated with a distance cutoff of 5 Å. FCC shows how many CC1α1-CAD/SOAR contacts, out of those present at time t_{ref}, are present at time t. **WT**: The all-to-all RMSD map indicates several reversible conformational transitions (orange stripes), in line with relatively large fluctuations of the CAD/SOAR RMSD around its equilibrium value. This is also reflected in the strongly fluctuating IE for CC1α1-CAD/SOAR interactions. These fluctuations can be associated with numerous electrostatic interaction partners in CC1α1 and CAD/SOAR, respectively, whose electrostatic interaction energy spreads by more than 20 kcal/mol around their respective mean values (Supp. Figure 10). The FCC map finally reveals five distinct shifts in the CC1α1-CAD/SOAR binding interface (dark vertical stripes). Notably though, these shifts are reversible, as indicated by high FCC around (t_{ref}, t) = (0 ns, 500 ns). **F394D**: All-to-all RMSD and CAD/SOAR RMSD suggest several smaller, reversible transitions and one larger one toward the very end of the 1 µs run. Closer analysis shows that these transitions primarily concern CC1α2,3, which are reported to be highly flexible in single molecule experiments [1]. CC1α1-CAD/SOAR IE and the number of contacts are rather stable. The FCC map shows that a shift in the CC1α1-CAD/SOAR binding interface occurs at around t = 250 ns. As indicated by the bottom right corner of the FCC map, about 65% of CC1α1-CAD/SOAR contacts in the initial and final conformations, respectively, are identical. Their overall number increases by about 20%. **F394K**: All indicators remain stable throughout the entire simulation.

Supplementary Figure 2 B:

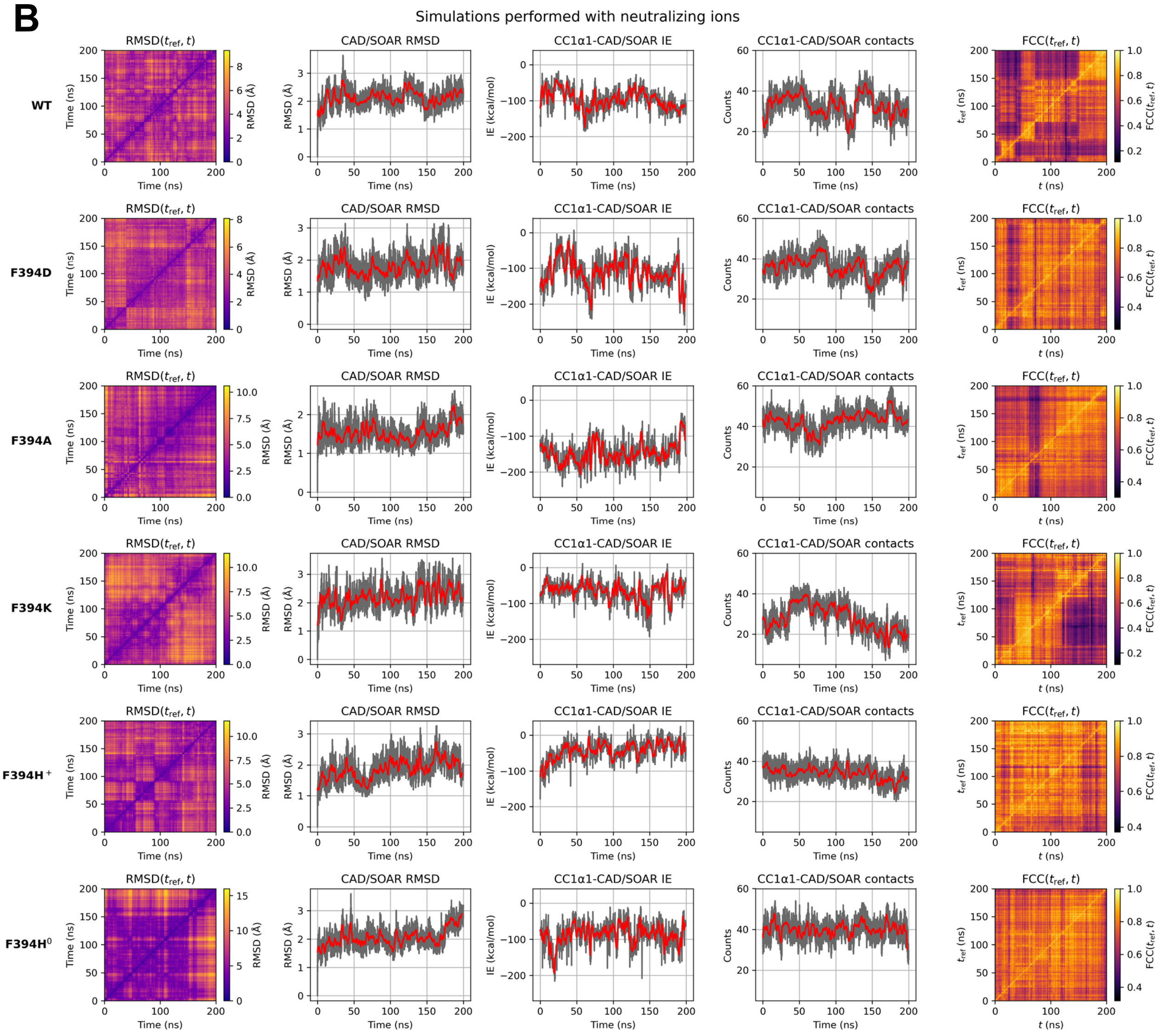

Structural stability

Structural stability for the final 200 ns of simulations of F394X (X=A,H,F,D,K) was performed in neutralized simulation boxes. The plots can be interpreted as follows. **WT**: RMSD and interaction energy are stable throughout the simulation. The FCC map reveals some rearrangement in the CC1α1-CAD/SOAR binding interface. Comparing the initial and final states, their respective binding interfaces still show ≈ 80% overlap. **F394D**: All-to-all RMSD reveals some conformational transitions of the overall protein, but CAD/SOAR RMSD and particularly the FCC remain very stable throughout the entire simulation. **F394A**: All indicators remain stable. A single decrease in the FCC map is associated with a short fluctuation in the number of CC1α1-CAD/SOAR contacts. **F394K**: All-to-all RMSD implies a transition at ≈ 110 ns, yet CAD/SOAR RMSD remains constant throughout. FCC implies that CC1α1-CAD/SOAR binding partners hardly change, but the number of binding partners

is subject to some fluctuation. **F394H$^+$**: All indicators remain stable throughout the simulation. **F394H^0**: The two RMSD plots imply a conformational transition towards the end of the run. All other indicators remain stable throughout the simulation.

Supplementary Figure 3:

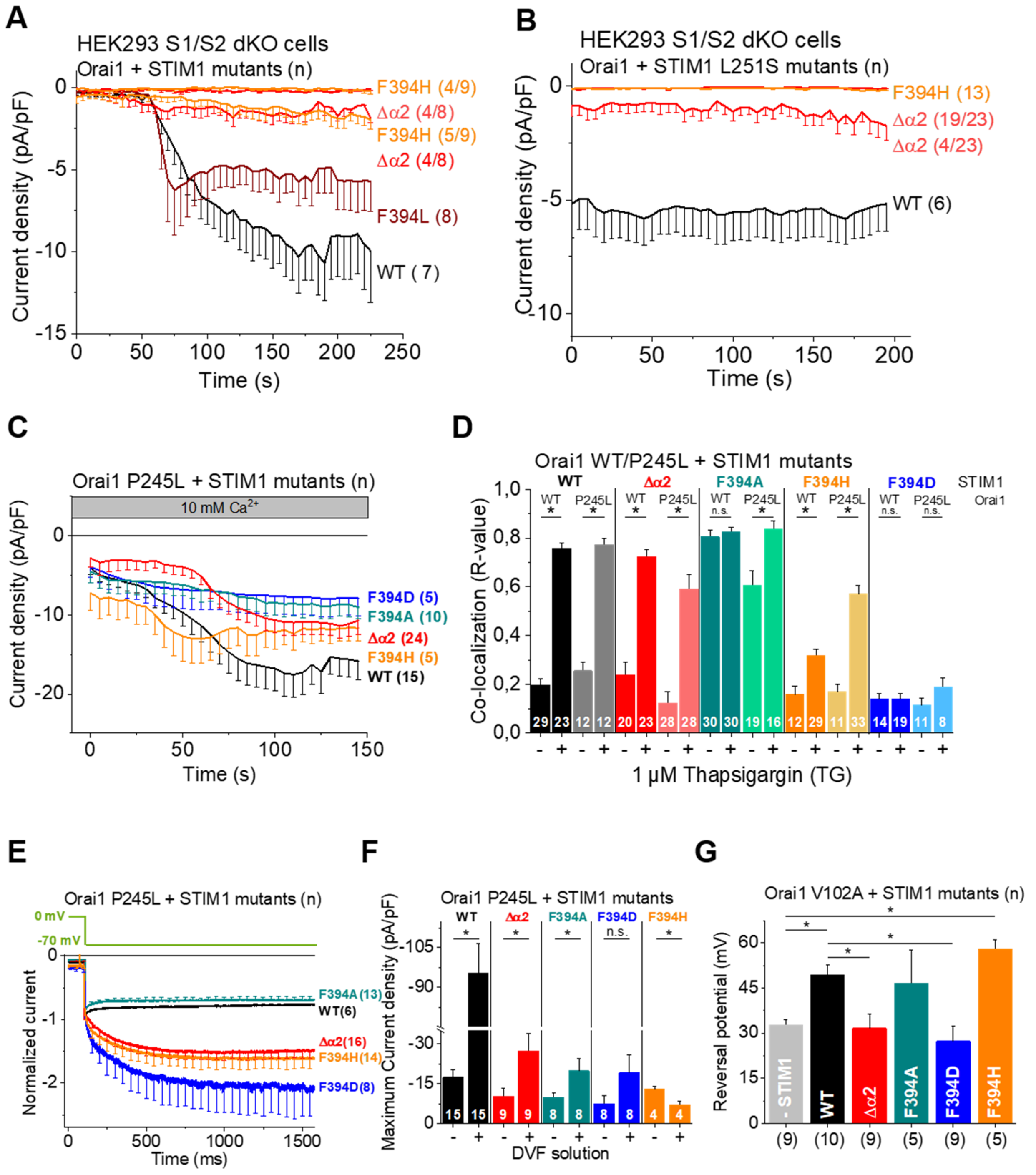

CRAC channel hallmarks of STIM1 SOARα2 mutants

(**A**) Time course of whole cell inward currents at -74 mV of STIM1/STIM2 dKO HEK293 cells co-expressing Orai1 together with the following STIM1 constructs: STIM1 WT, Δα2 (Δaa 393-398), F394H and F394L. (**B**) Time course of whole cell inward currents at -74 mV of STIM1/STIM2 dKO

HEK293 cells co-expressing Orai1 together with the following STIM1 constructs: STIM1 L251S WT, Δα2 (Δaa 393-398), and F394H. **(C)** Time course of whole cell inward currents at -74 mV of HEK293 cells co-expressing Orai1 P245L together with the following STIM1 constructs: STIM1 WT, Δα2 (Δaa 393-398), F394A, F394D and F394H. **(D)** Calculated Pearson correlation coefficient (R-value) as a quantitative measurement of co-localization between the indicated CFP-STIM1 mutants and YFP-Orai1 WT or YFP-Orai1 P245L before (-) and after (+) treatment with 1 µM TG. Darker and lighter color variants indicate measurements using Orai1 WT and P245L, respectively. The number n of cells is indicated within each bar. **(E)** Fast calcium-dependent inactivation (FCDI) measurements of Orai1 P245L and STIM1 using the same constructs as in (C) obtained upon application of a voltage step to -70 mV from a holding potential of 0 mV (indicated by green step). **(F)** Comparison of the maximum current density of Orai1 P245L and STIM1 constructs (same as in (C)) before (-) and after (+) change to Na^+-containing DVF solution. **(G)** Comparison of the reversal potentials of Orai1 V102A alone (-STIM1) and Orai1 V102A co-expressed with the same STIM1 constructs as in (C). Data represent mean values ± SEM. HEK293 cells were used for all experiments. Student's two-tailed t-test was employed for statistical analyses with differences considered statistically significant at $p < 0.05$. Asterisks (*) indicate significant difference. Color code: WT (black), Δα2 (red), F394A (dark cyan), F394D (blue), F394H (orange).

Supplementary Figure 4:

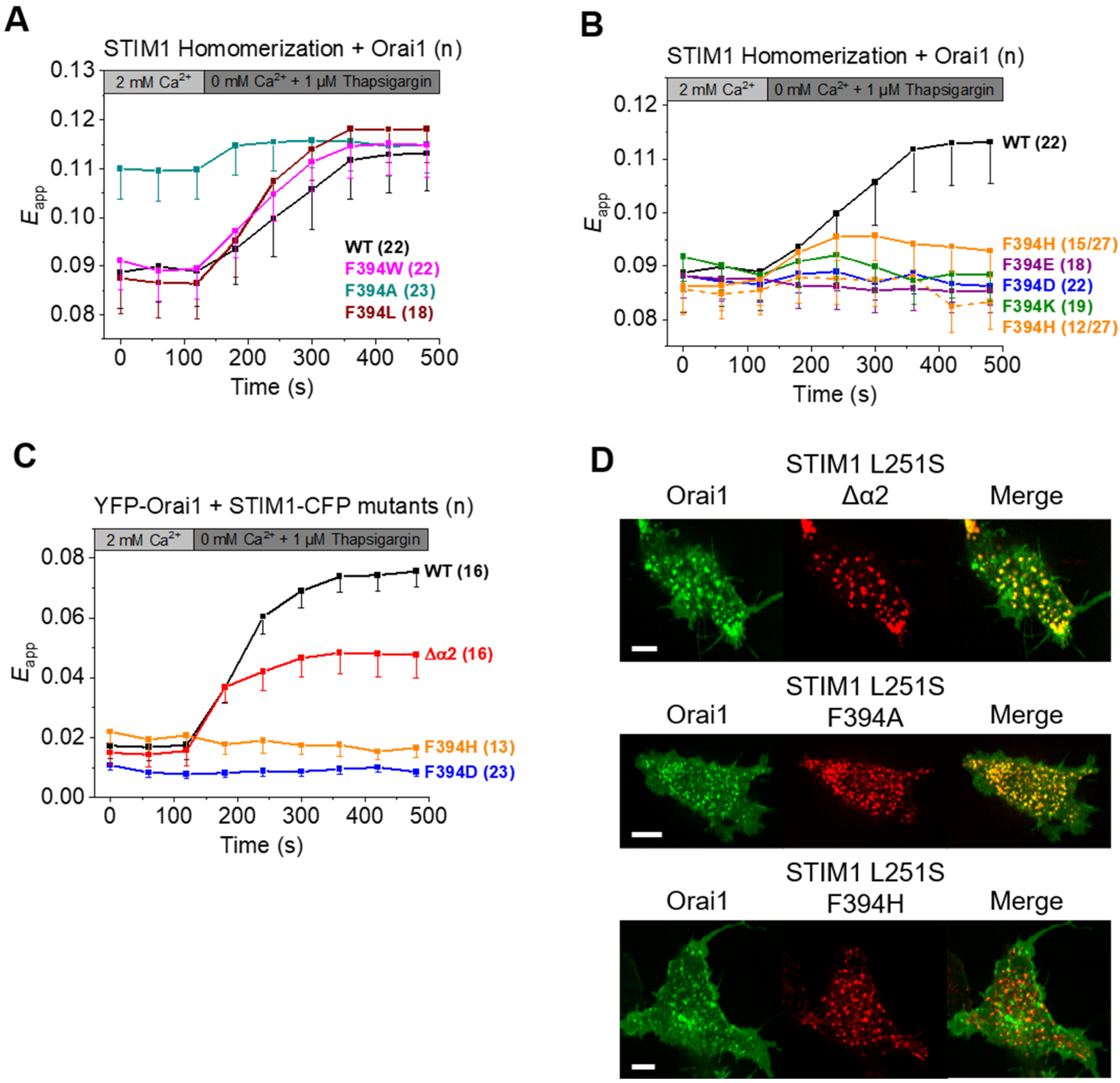

Homomerization and puncta formation of STIM1 α2 mutants

(A) (B) Time course of FRET (E_{app}) monitoring the homomerization of the following CFP/YFP-labeled STIM1 constructs co-expressed with Orai1 WT in response to 1 µM TG: STIM1 WT, F394A, F394D, F394E, F394H, F394K, F394L, and F394W. **(C)** Time course of FRET (E_{app}) monitoring the interaction of YFP-Orai1 with STIM1-CFP proteins, including STIM1 WT, STIM1 Δα2, STIM1 F394H, and STIM1 F394D co-expressed with Orai1 WT in response to 1 µM TG. **(D)** Confocal fluorescence images of CFP-Orai1 WT and YFP-STIM1 L251S Δα2 (Δaa 393-398), L251S F394A, and L251S F394H (CFP in green, YFP in red) as well as an overlay (Merge in yellow) for visual comparison. The length of scale bars corresponds to 5 µm. Data represent mean values ± SEM. HEK293 cells were used for all experiments. Color code: WT (black), F394A (dark cyan), F394D (blue), F394E (purple), F394H (orange), F394K (green), F394L (brown), F394W (magenta).

Supplementary Figure 5:

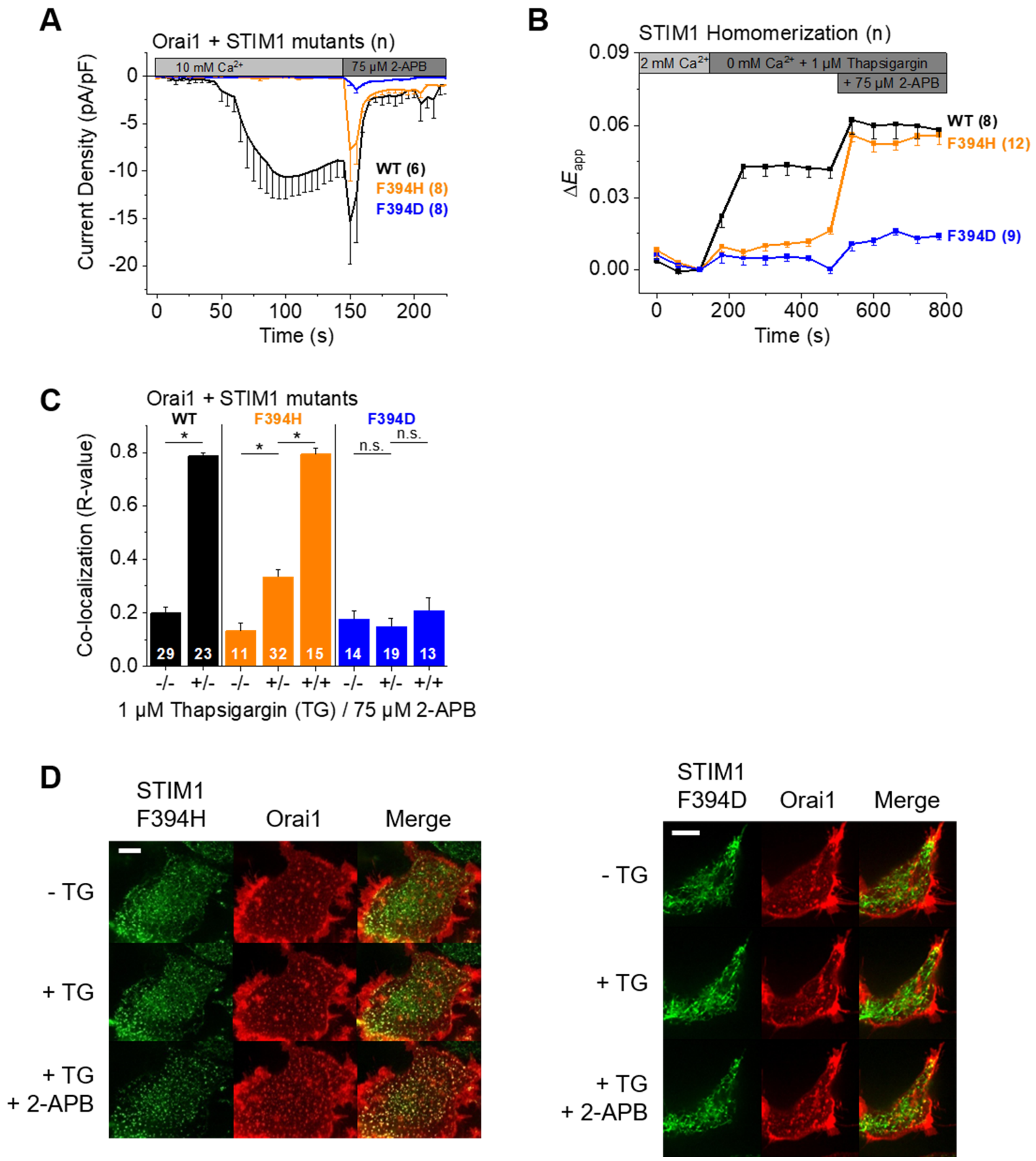

2-APB is able to compensate for the loss of homomerization of STIM1 F394H but not of STIM1 F394D

(A) Time course of whole cell inward currents at -74 mV activated by passive store depletion of HEK293 cells co-expressing Orai1 WT together with the following STIM1 constructs: STIM1 WT,

F394D, and F394H. The cells were treated with 75 µM 2-APB after 145 seconds. **(B)** Time course of FRET (E_{app}) monitoring the homomerization of the respective CFP/YFP-labeled STIM1 constructs specified in (A) in response to 1 µM TG and 75 µM 2-APB. **(C)** Calculated Pearson correlation coefficient (R-value) as a quantitative measurement of co-localization between the indicated CFP-STIM1 constructs (same as in (A)) and YFP-Orai1 WT before (-/-) and after treatment with 1 µM TG (+/-) as well as 75 µM 2-APB (+/+). The number n of cells is indicated within each bar. **(D)** Confocal fluorescence images of CFP-STIM1 F394D/F394H and YFP-Orai1 WT (CFP in green, YFP in red) before and after treatment with 1 µM TG/75 µM 2-APB as well as an overlay (Merge in yellow) for visual comparison. The length of scale bars corresponds to 5 µm. Images were captured in the periphery of the cells. Data represent mean values ± SEM. HEK293 cells were used for all experiments. Student's two-tailed t-test was employed for statistical analyses with differences considered statistically significant at $p < 0.05$. Asterisks (*) indicate significant difference. Color code: WT (black), F394D (blue), F394H (orange).

Supplementary Figure 6:

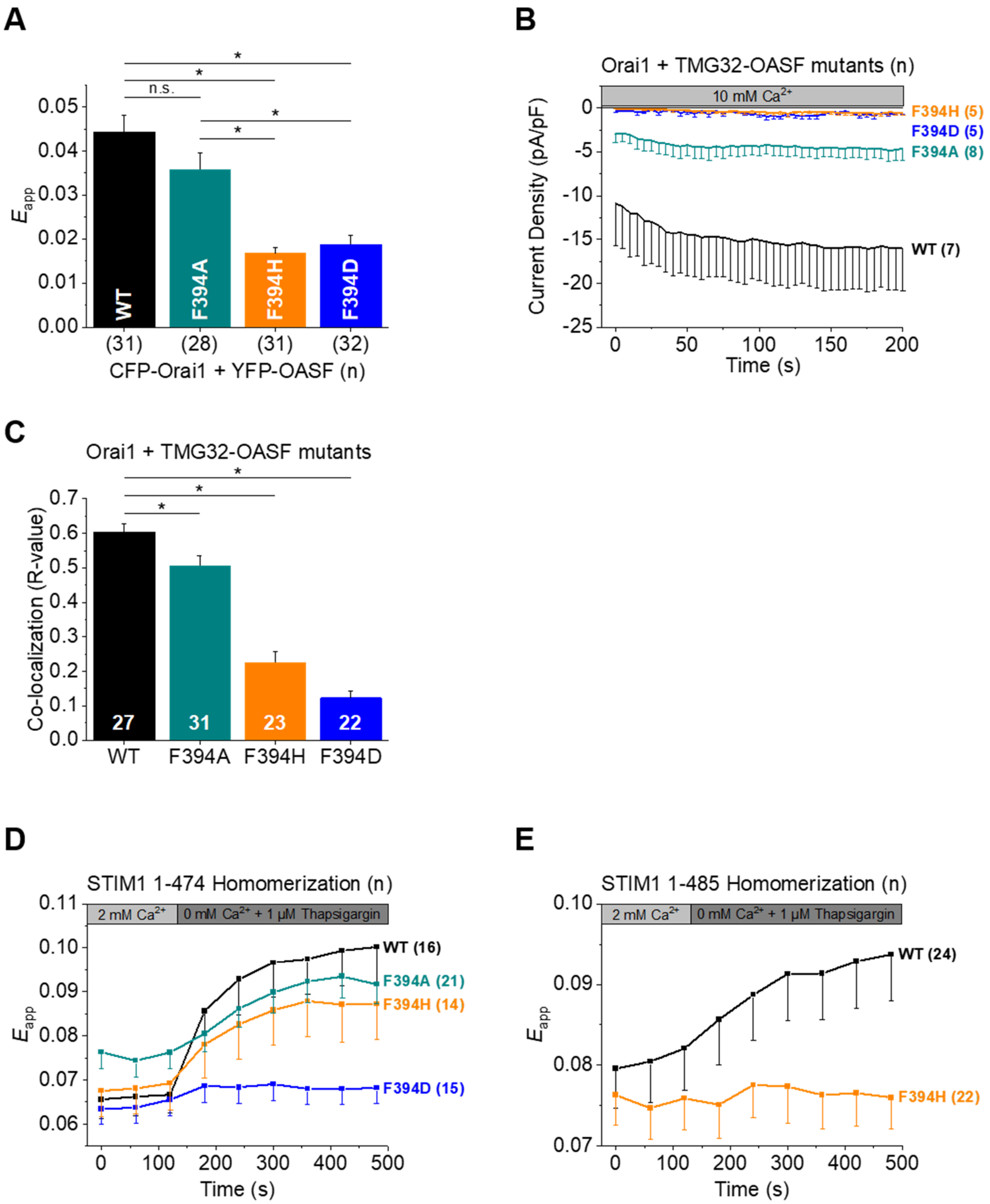

Effects of cytosolic and membrane-anchored OASF mutants on the activation of and co-localization with Orai1

(A) Intermolecular FRET (E_{app}) measurements of CFP-Orai1 and YFP-OASF fragments including: OASF WT, F394A, F394H, and F394D. **(B)** Time course of whole cell inward currents at -74 mV of HEK293 cells co-expressing Orai1 WT together with the following TMG32-OASF constructs: TMG32-OASF WT, F394A, F394D, and F394H. **(C)** Calculated Pearson correlation coefficient (R-value) as a quantitative measurement of co-localization between the same TMG32-OASF constructs as in (A) and Orai1 WT. **(D)** Time course of FRET (E_{app}) monitoring the homomerization of CFP/YFP-labeled STIM1 1-474 constructs in response to 1 µM TG using OASF WT, F394A, F394H, and F394D. **(E)** Time course of FRET (E_{app}) monitoring the homomerization of CFP/YFP-labeled STIM1 1-485 constructs in response to 1 µM TG using OASF WT and F394H. The number n of cells is indicated within each bar. Data represent mean values ± SEM. HEK293 cells were used for all experiments. Student's two-tailed t-test was employed for statistical analyses with differences considered statistically significant at $p < 0.05$. Asterisks (*) indicate significant difference. Color code: WT (black), F394A (dark cyan), F394D (blue), F394H (orange).

Supplementary Figure 7:

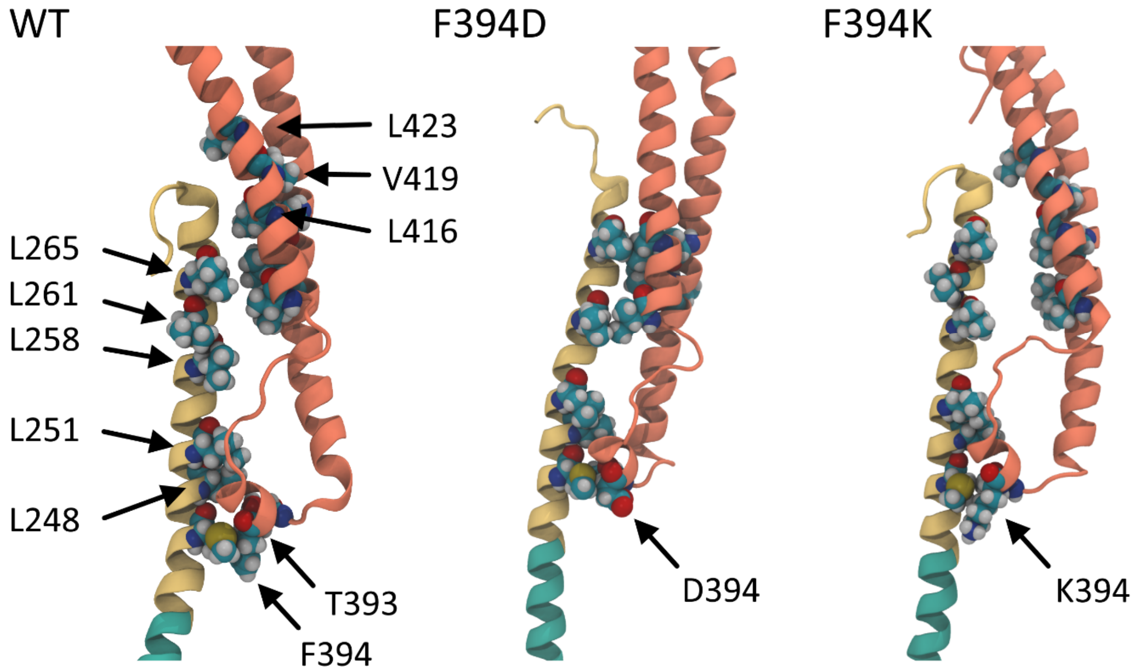

CC1α1-CAD/SOAR binding interface comparison

CC1α1-CAD/SOAR binding interface obtained from MD simulations for the WT, F394D and F394K, respectively. Interaction residues reported in reference [2], as well as F/D/K394, are shown in space-filling representation. The number of CC1α1-CAD/SOAR contacts for the respective frames shown here are 47, 49 and 34 for the WT, F394D and F394K, respectively.

Supplementary Figure 8:

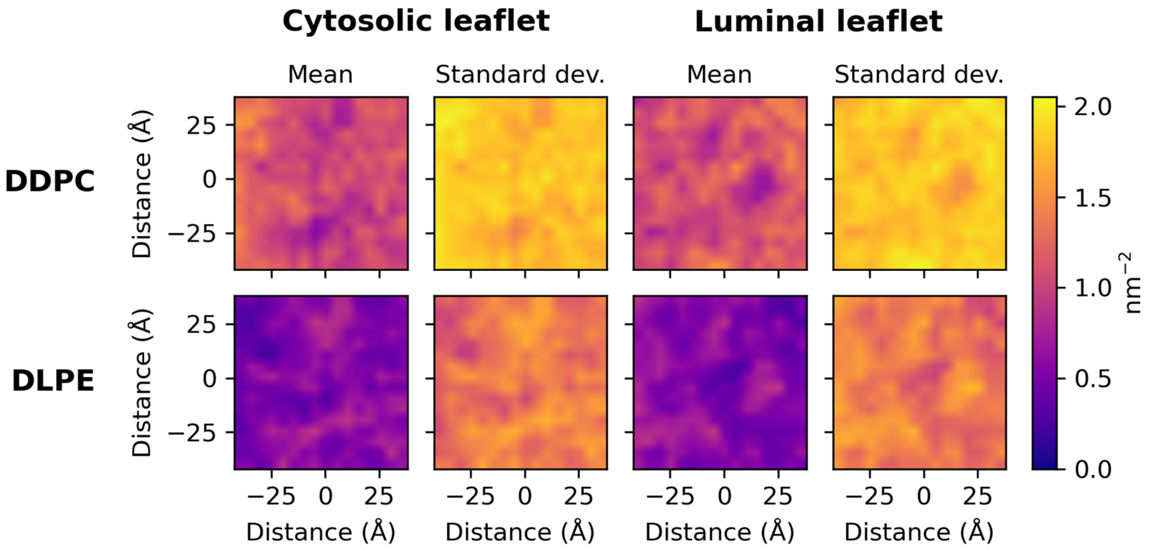

Lipid distribution

Lipid distribution of the two lipid species used (DDPC, DLPE) in the cytosolic and luminal leaflets, respectively. Lipid positions were taken from the final 500 ns of our WT simulation performed at 0.15 M KCl.

Supplementary Figure 9:

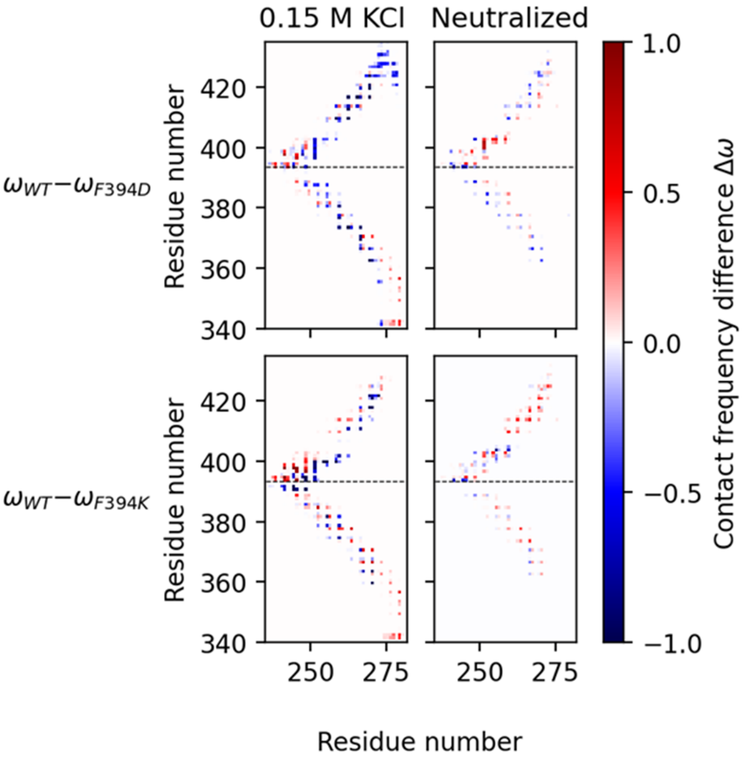

Contact difference maps

Contact difference maps for the WT, F394D and F394K, simulated with 0.15 M KCl and with neutralized simulation boxes, respectively. ω_{F394X}, X = F,D,K, denotes the contact frequency of contacts between residues in CC1α1 and CAD/SOAR, i.e. the fraction of simulation time during which a contact is formed. Contacts were calculated with a distance cutoff of 4.5 Å. The difference between the contact frequency calculated for the WT and the contact frequency of F394D (F394K) is shown in the upper (lower) panel. Red (blue) colors indicate that a contact is more prevalent in the WT (mutant). Adjacent red and blue dots in the map are indicative of a shift in the binding interface.

Supplementary Figure 10:

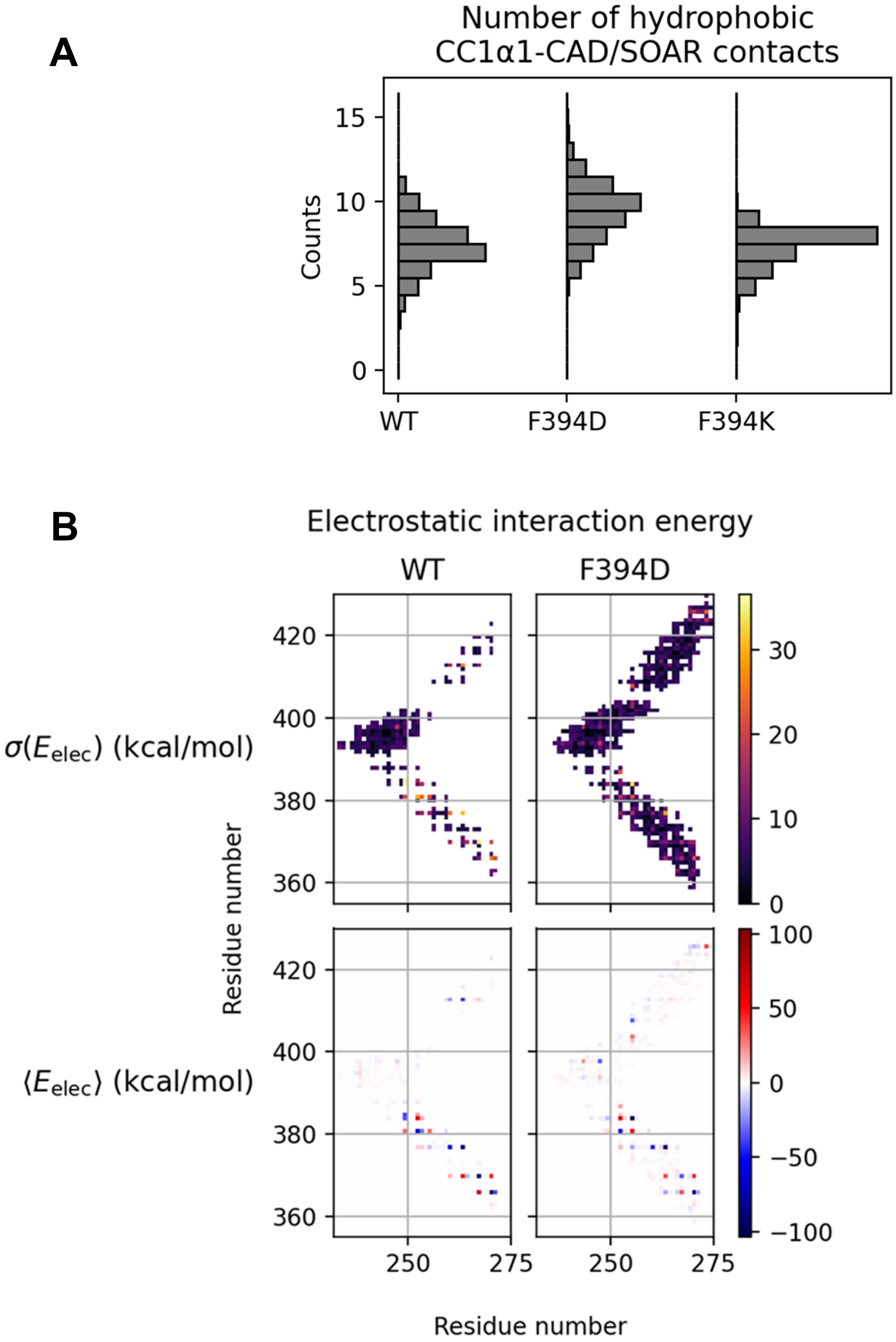

Number of hydrophobic contacts and electrostatic interaction energy

(A) Distribution of the number of hydrophobic contacts between CC1α1 and CAD/SOAR. Contacts were calculated with a distance cutoff of 5 Å between hydrophobic residues. **(B)** Standard deviation

(σ) and mean (⟨·⟩) of the electrostatic interaction energy E_{elec} for interactions between residues in CC1α1 and CAD/SOAR, respectively.

Supplementary Figure 11:

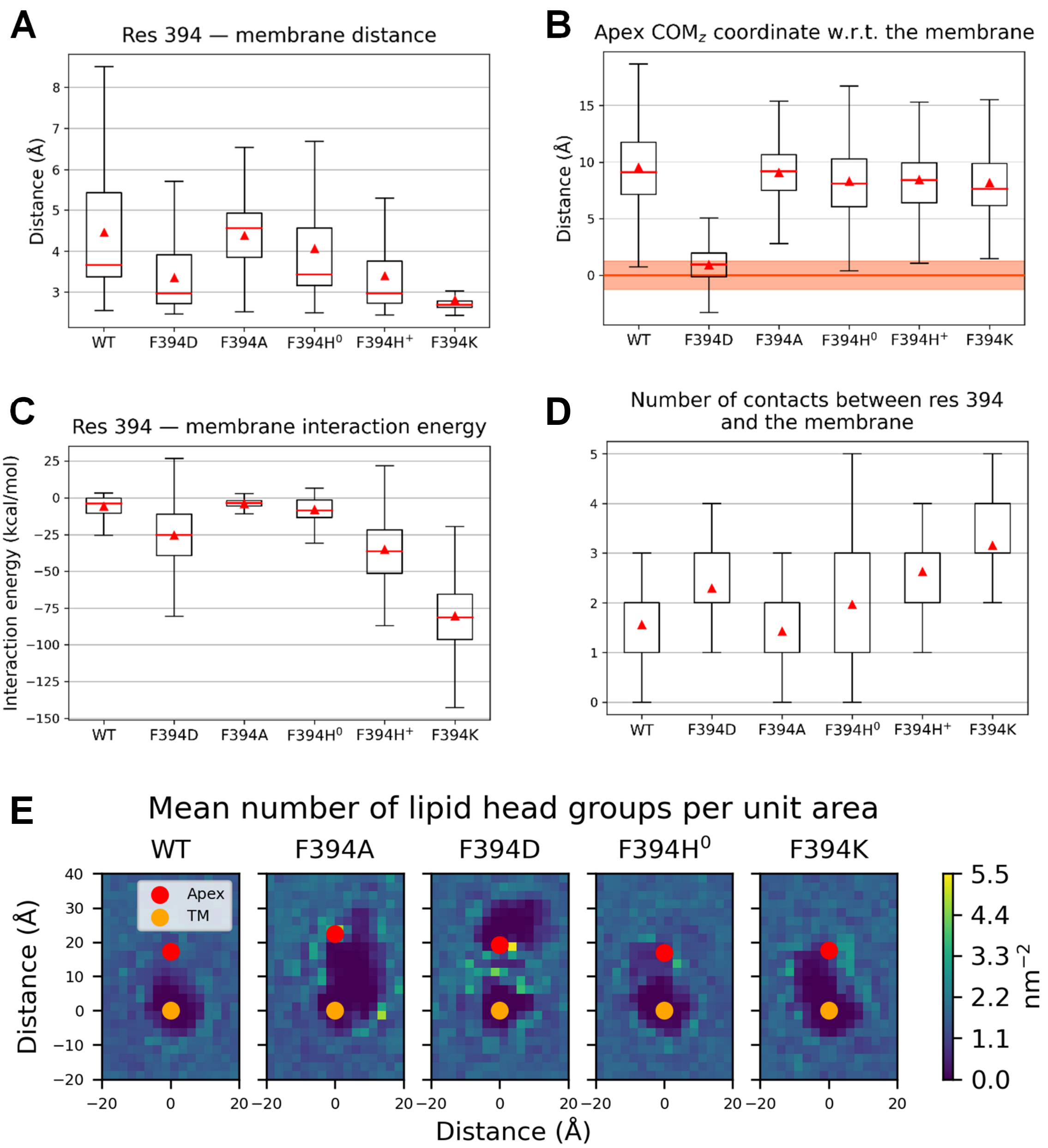

X394-membrane interaction

Data obtained from simulations of F394X (X= F,D,A,H,K) performed using neutralized simulation boxes. **(A)** Closest interatomic distance between the membrane and X394, X=F,D,A,H,K. Mean (median) values are denoted by a red triangle (line). H^0 refers to a neutral histidine with a hydrogen at the epsilon nitrogen, while H^+ refers to a positively charged histidine carrying both a delta and an epsilon hydrogen. **(B)** Box plots displaying the *z* coordinate of the STIM1 α2 apex center of mass (COM) relative to the ER membrane surface at z = 0 Å. The approximate thickness of the layer comprised of lipid headgroups is indicated by an orange stripe. Mean (median) values are denoted by

a red triangle (line). **(C)** Interaction energies for interactions between the membrane and F/A/D/H/K394. The interaction energy comprises electrostatic and van der Waals energy. **(D)** Number of contacts between F/D/A/H/K394 and the ER membrane. Mean values are denoted by a red triangle. **(E)** Mean number of lipid head groups in the cytosolic ER membrane leaflet per unit area (nm^2) in the membrane plane. Red and orange dots indicate the positions of the CAD/SOAR apex and the TM domain, respectively. For the case of F394D, the density map shows that this mutation leads to the CAD/SOAR apex partitioning into the membrane, as also indicated in panel B.

Supplementary Figure 12:

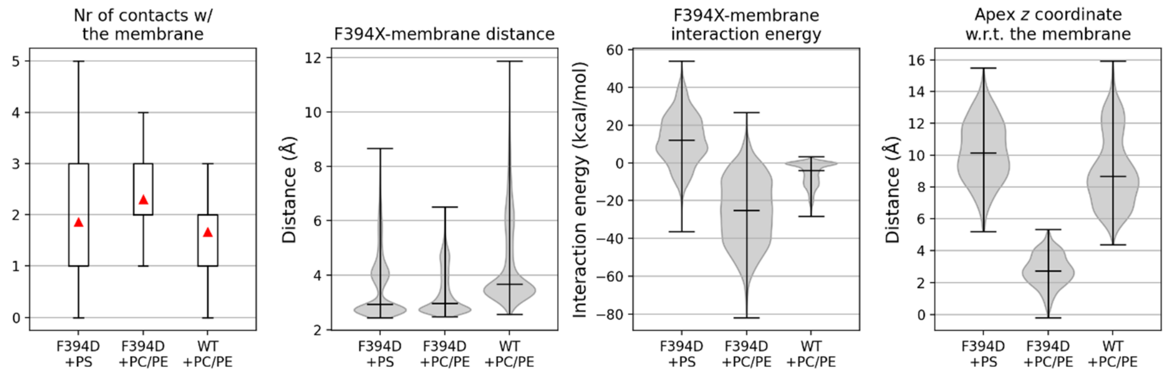

F394D/WT for different lipid composition

Data were obtained from a simulation of F394D embedded in a negatively charged phosphatidylserine (PS) membrane. For comparison, data for F394D/WT in the PS/PE membrane (cf. Supp. Figure 11 A-D) is also shown. Mean (median) values are denoted by a red triangle (line).

Supplementary Figure 13:

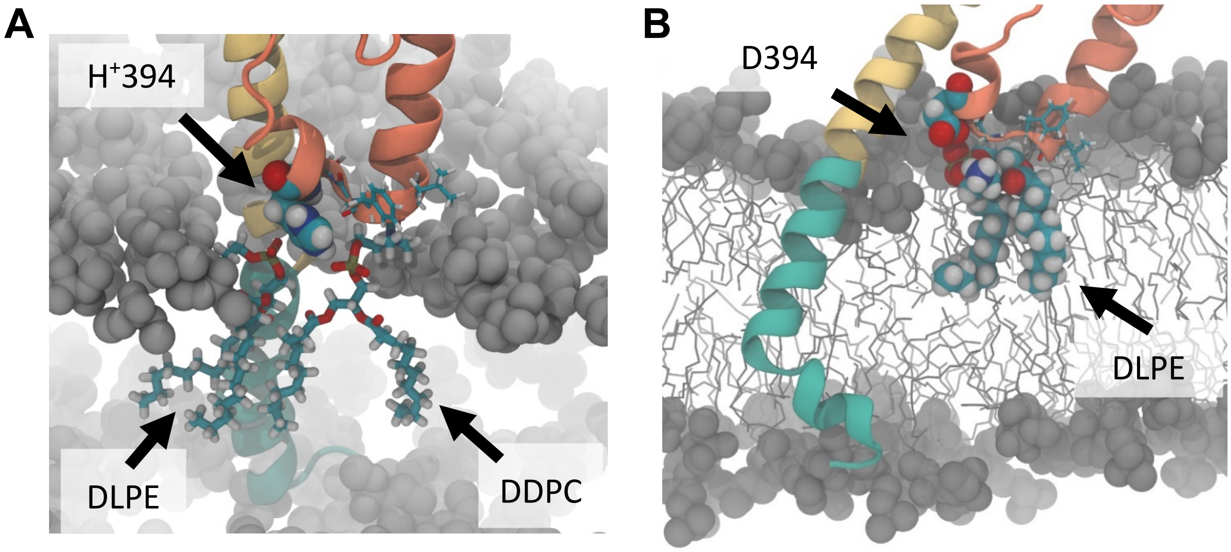

F394H⁺/D and bound lipids

A close-up view of lipids binding to $H^{+}394$ **(A)** and D394 **(B)**. **(A)** At the selected frame $H^{+}394$ forms contacts with two lipids (DPLE and DDPC). **(B)** In our simulation of F394D using neutralizing ions, D394 forms contacts with the shown DLPE molecule throughout 60% of the simulation time.

Supplementary Figure 14:

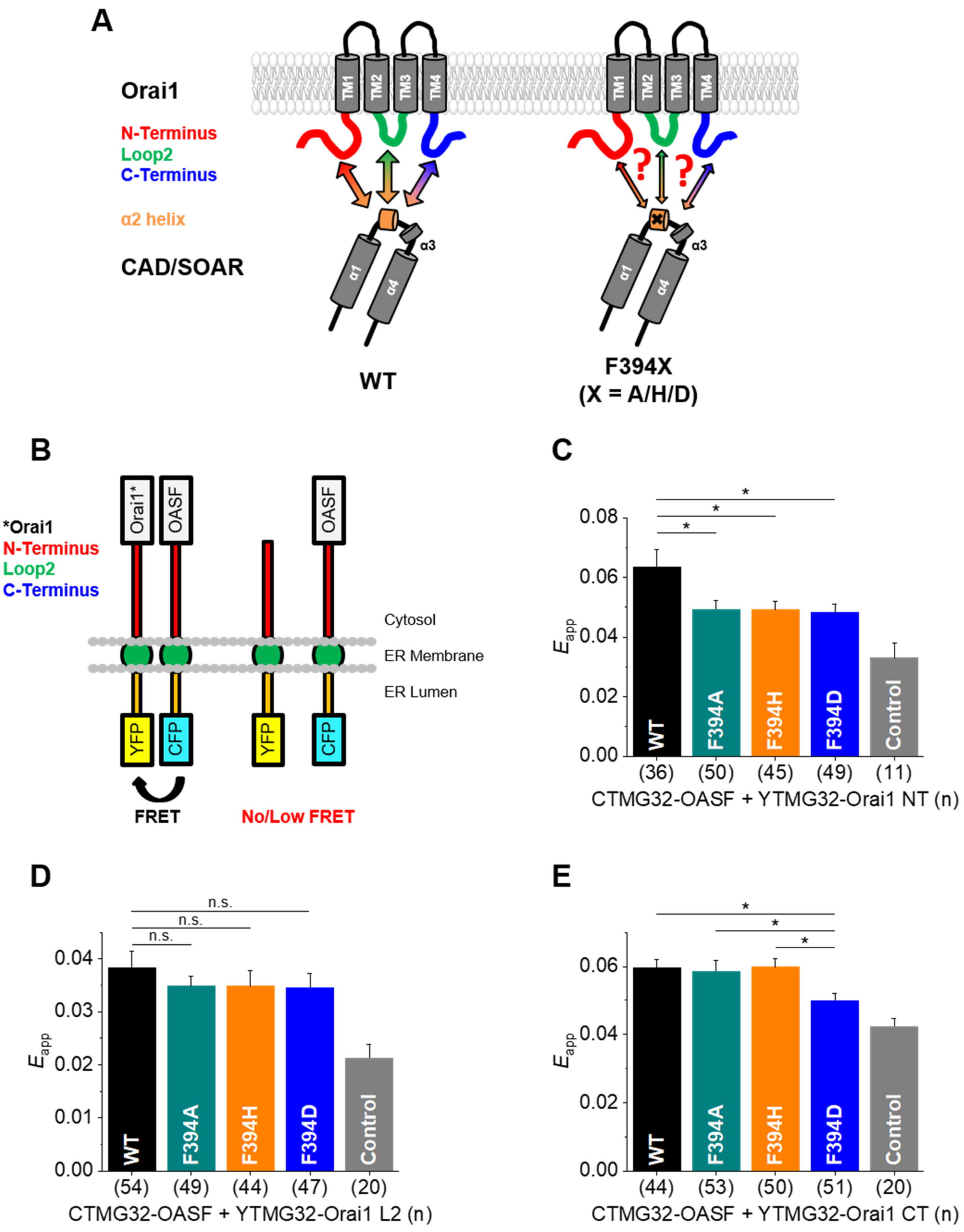

Mutation of F394 in OASF interferes with the coupling to one of the Orai1 cytosolic fragments
(A) Simplified representation of potential interaction partners of CAD/SOAR within Orai1, which include the Orai1 N-terminus, Loop2, and C-terminus (left). Mutations of F394 in the α2 region seem to affect the coupling to Orai1 (right). **(B)** Cartoon representation of the FIRE system (*cf.* also Figure 4F) showing interaction of CFP-TMG32-OASF (CTMG32-OASF) with YFP-TMG32-Orai1 (YTMG32-Orai1) fragments: N-terminus (NT), Loop2 (L2), or C-terminus (CT). A control experiment lacking FIRE interaction is indicated on the right. **(C)** Intermolecular FIRE (E_{app}) interaction measurements of ER membrane-bound CTMG32-OASF + YTMG32-Orai1 NT using the following OASF constructs: OASF WT, F394A, F394D, and F394H. **(D)** Same as in (C) but for CTMG32-OASF + YTMG32-Orai1 L2. **(E)** Same as in (C) but for CTMG32-OASF + YTMG32-Orai1 CT. For the respective control measurements, CTMG32-OASF was co-expressed with a YTMG32 construct lacking an Orai1 fragment. Control E_{app} is significantly smaller than all tested interactions in all three cases (NT, L2, and CT; not indicated). Data represent mean values ± SEM. HEK293 cells were used for all experiments. Student's two-tailed t-test was employed for statistical analyses with differences considered statistically significant at $p < 0.05$. Asterisks (*) indicate significant difference. Color code: WT (black), F394A (dark cyan), F394D (blue), F394H (orange), Control (gray).

References:

1. van Dorp S, Qiu R, Choi UB, Wu MM, Yen M, Kirmiz M, Brunger AT, Lewis RS (2021) Conformational dynamics of auto-inhibition in the ER calcium sensor STIM1. https://wwwbiorxivorg/content/101101/20201217423361v1

2. Ma G, Wei M, He L, Liu C, Wu B, Zhang SL, Jing J, Liang X, Senes A, Tan P, Li S, Sun A, Bi Y, Zhong L, Si H, Shen Y, Li M, Lee MS, Zhou W, Wang J, Wang Y, Zhou Y (2015) Inside-out Ca(2+) signalling prompted by STIM1 conformational switch. Nat Commun 6:7826. doi:10.1038/ncomms8826
